# Development and validation of a gonadotropin dose selection model for optimized ovarian stimulation in IVF/ICSI: an individual participant data meta-analysis

**DOI:** 10.1093/humupd/dmae032

**Published:** 2024-12-20

**Authors:** Nienke Schouten, Rui Wang, Helen Torrance, Theodora Van Tilborg, Ercan Bastu, Christina Bergh, Thomas D’Hooghe, Jesper Friis Petersen, Kannamannadiar Jayaprakasan, Yacoub Khalaf, Ellen Klinkert, Antonio La Marca, Lan Vuong, Louise Lapensée, Sarah Lensen, Åsa Magnusson, Adolfo Allegra, Anders Nyboe Andersen, Simone Oudshoorn, Biljana Popovic-Todorovic, Ben Willem Mol, Marinus Eijkemans, Frank Broekmans

**Affiliations:** Division Woman and Baby, Reproductive Medicine, University Medical Center Utrecht, University of Utrecht, Utrecht, The Netherlands; Department of Obstetrics and Gynaecology, Monash Medical Centre, Monash University, Clayton, VIC, Australia; Division Woman and Baby, Reproductive Medicine, University Medical Center Utrecht, University of Utrecht, Utrecht, The Netherlands; Division Woman and Baby, Reproductive Medicine, University Medical Center Utrecht, University of Utrecht, Utrecht, The Netherlands; Acibadem University Faculty of Medicine, Department of Obstetrics and Gynecology, Istanbul University School of Medicine, Istanbul, Turkey; Department of Obstetrics and Gynaecology, Institute of Clinical Sciences, Sahlgrenska Academy, Sahlgrenska University Hospital, Gothenburg University, Gothenburg, Sweden; Global Medical Affairs, Research and Development, Merck Healthcare KGaA, Darmstadt, Germany; Department of Obstetrics and Gynecology, North Zealand Hospital, Hilleroed, Denmark; Royal Derby Hospital, Derby & University of Nottingham, Nottingham, UK; Assisted Conception Unit, Guy’s & St Thomas’s Hospital, London, UK; Department of Obstetrics & Gynaecology, University Medical Center Groningen, Groningen, The Netherlands; Department of Obstetrics Gynaecology and Paediatric Sciences, University of Modena and Reggio Emilia, Modena, Italy; Department of Obstetrics and Gynecology, University of Medicine and Pharmacy at Ho Chi Minh City, Ho Chi Minh City, Vietnam; Reproductive Endocrinology and Infertility, Clinique ovo, Montréal, Canada; Department of Obstetrics and Gynaecology, Royal Women’s Hospital, University of Melbourne, Melbourne, Victoria, Australia; Department of Obstetrics and Gynaecology, Institute of Clinical Sciences, Sahlgrenska Academy, Sahlgrenska University Hospital, Gothenburg University, Gothenburg, Sweden; Reproductive Medicine Unit, ANDROS Day Surgery Clinic, Palermo, Italy; The Fertility Department, Copenhagen University Hospital, Rigshospitalet, Copenhagen, Denmark; Division Woman and Baby, Reproductive Medicine, University Medical Center Utrecht, University of Utrecht, Utrecht, The Netherlands; Center for Reproductive Medicine, Universitair Ziekenhuis Brussel, Brussels, Belgium; Department of Obstetrics and Gynaecology, Monash Medical Centre, Monash University, Clayton, VIC, Australia; Division Woman and Baby, Reproductive Medicine, University Medical Center Utrecht, University of Utrecht, Utrecht, The Netherlands; Division Woman and Baby, Reproductive Medicine, University Medical Center Utrecht, University of Utrecht, Utrecht, The Netherlands

**Keywords:** ovarian stimulation, individualized dosing, gonadotropin starting dose, IPD-MA, prediction model

## Abstract

**BACKGROUND:**

The ovarian response to gonadotropin stimulation varies widely among women, and could impact the probability of live birth as well as treatment risks. Many studies have evaluated the impact of different gonadotropin starting doses, mainly based on predictive variables like ovarian reserve tests (ORT) including anti-Müllerian hormone (AMH), antral follicle count (AFC), and basal follicle-stimulating hormone (bFSH). A Cochrane systematic review revealed that individualizing the gonadotropin starting dose does not affect efficacy in terms of ongoing pregnancy/live birth rates, but may reduce treatment risks such as the development of ovarian hyperstimulation syndrome (OHSS). An individual patient data meta-analysis (IPD-MA) offers a unique opportunity to develop and validate a universal prediction model to help choose the optimal gonadotropin starting dose to minimize treatment risks without affecting efficacy.

**OBJECTIVE AND RATIONALE:**

The objective of this IPD-MA is to develop and validate a gonadotropin dose-selection model to guide the choice of a gonadotropin starting dose in IVF/ICSI, with the purpose of minimizing treatment risks without compromising live birth rates.

**SEARCH METHODS:**

Electronic databases including MEDLINE, EMBASE, and CRSO were searched to identify eligible studies. The last search was performed on 13 July 2022. Randomized controlled trials (RCTs) were included if they compared different doses of gonadotropins in women undergoing IVF/ICSI, presented at least one type of ORT, and reported on live birth or ongoing pregnancy. Authors of eligible studies were contacted to share their individual participant data (IPD). IPD and information within publications were used to determine the risk of bias. Generalized linear mixed multilevel models were applied for predictor selection and model development.

**OUTCOMES:**

A total of 14 RCTs with data of 3455 participants were included. After extensive modeling, women aged 39 years and over were excluded, which resulted in the definitive inclusion of 2907 women. The optimal prediction model for live birth included six predictors: age, gonadotropin starting dose, body mass index, AFC, IVF/ICSI, and AMH. This model had an area under the curve (AUC) of 0.557 (95% confidence interval (CI) from 0.536 to 0.577). The clinically feasible live birth model included age, starting dose, and AMH and had an AUC of 0.554 (95% CI from 0.530 to 0.578). Two models were selected as the optimal model for combined treatment risk, as their performance was equal. One included age, starting dose, AMH, and bFSH; the other also included gonadotropin-releasing hormone (GnRH) analog. The AUCs for both models were 0.769 (95% CI from 0.729 to 0.809). The clinically feasible model for combined treatment risk included age, starting dose, AMH, and GnRH analog, and had an AUC of 0.748 (95% CI from 0.709 to 0.787).

**WIDER IMPLICATIONS:**

The aim of this study was to create a model including patient characteristics whereby gonadotropin starting dose was predictive of both live birth and treatment risks. The model performed poorly on predicting live birth by modifying the FSH starting dose. On the contrary, predicting treatment risks in terms of OHSS occurrence and management by modifying the gonadotropin starting dose was adequate. This dose-selection model, consisting of easily obtainable patient characteristics, aids in the choice of the optimal gonadotropin starting dose for each individual patient to lower treatment risks and potentially reduce treatment costs.

## Graphical abstract

**Figure:**
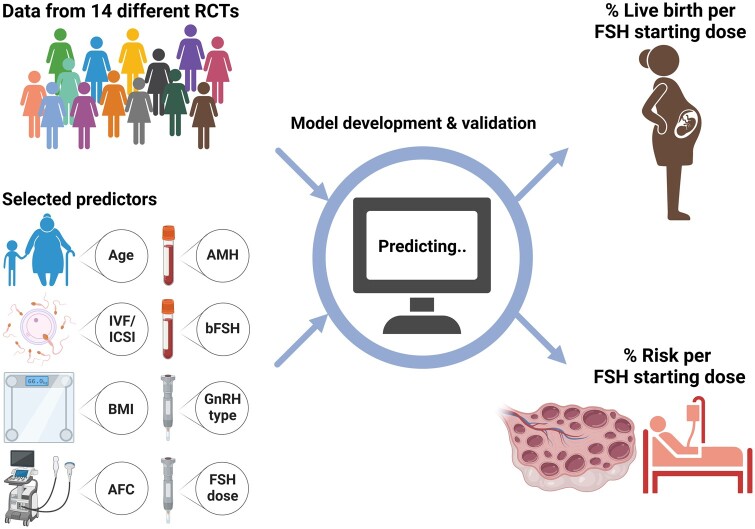
This IPD-MA aimed to develop a model predicting live birth and treatment risks in IVF/ICSI for different gonadotropin starting doses; the model performed poorly in predicting live birth but effectively assessed treatment risks, offering a possible tool for individualized dose selection.

## Introduction

Since the introduction of *in vitro* fertilization (IVF) and intracytoplasmic sperm injection (ICSI), many studies have focused on improving ovarian stimulation protocols. Many strategies have been proposed to improve live birth rates and minimize treatment risks, one of them being individualized dosing. The ovarian response to gonadotropin stimulation varies widely among individuals and this may impact both live birth rates and treatment risks. Therefore, previous studies have evaluated the impact of different gonadotropin starting doses on both treatment risks and live birth rates, often based on predictive variables like ovarian reserve tests (ORTs) (Lensen *et al.*, 2024; Janse *et al.*, 2021).

An important treatment risk is the development of moderate or severe ovarian hyperstimulation syndrome (OHSS). The incidence of OHSS ranges between ∼0.2% and 9% and is higher in high-risk groups such as women with polycystic ovary syndrome (PCOS) (Papanikolaou *et al.*, 2006; Delvigne, 2009; Toftager *et al.*, 2016; Mourad *et al.*, 2017; Ishihara *et al.*, 2020; Wyns *et al.*, 2020; Kuroda *et al.*, 2021; Tomás *et al.*, 2021). OHSS is estimated to be fatal in 0.2 to 3 per 100 000 women undergoing ovarian stimulation for IVF/ICSI, so the importance of improving treatment safety cannot be sufficiently underlined (Brinsden *et al.*, 1995; Into Maternal CE, 2007; Braat *et al.*, 2010).

Many studies addressing ovarian stimulation approaches focus on the number of oocytes as an indicator of both live birth rates and treatment risks (Van der Gaast *et al.*, 2006; Sunkara *et al.*, 2011; Ji *et al.*, 2013; Steward *et al.*, 2014; Drakopoulos *et al.*, 2016; Vaughan *et al.*, 2017; Devesa *et al.*, 2018; Magnusson *et al.*, 2018; Law *et al.*, 2019). Sunkara was the first to suggest an association between the number of oocytes and live birth rates per fresh IVF cycle, demonstrating that among women undergoing standard gonadotropin dosing, the chance of having a live birth rises with an increasing number of oocytes up to 15 (Sunkara *et al.*, 2011). However, an extensive meta-analysis showed that increasing the gonadotropin starting dose in low or normal responders does not improve live birth rates, even though it results in a higher number of retrieved oocytes (Sterrenburg *et al.*, 2011; Lensen *et al.*, 2024). Additionally, randomized controlled trials (RCTs) applying cumulative live birth results from subsequent stimulation cycles as the outcome revealed no differences for individual dosing versus conventional dosing (Oudshoorn *et al.*, 2017; Van Tilborg *et al.*, 2017; Friis Petersen *et al.*, 2019).

On the other hand, varying the gonadotropin dosing clearly affects the incidence of OHSS in predicted hyper-responders, without compromising live birth rates (Andersen *et al.*, 2017; Oudshoorn *et al.*, 2017; Lensen *et al.*, 2024; Ishihara *et al.*, 2021; Janse, Eijkemans, and Fauser, 2021). As a higher number of growing follicles is associated with an increased risk of OHSS (Papanikolaou *et al.*, 2006; Jayaprakasan *et al.*, 2012; Steward *et al.*, 2014), a lower gonadotropin starting dose will result in a lower number of growing follicles, and a lower incidence of OHSS in predicted high responders (Oudshoorn *et al.*, 2017; Lensen *et al.*, 2024). However, one study showed a higher risk of cycle cancellation due to a poor response after receiving a lower gonadotropin starting dose in predicted hyper-responders.

Prediction models for live birth and treatment safety strategies based upon ORTs are included in the top 10 research priorities for infertility research (Duffy *et al.*, 2020). The studies reported above underline the potential of individualized gonadotropin dosing to improve treatment safety in IVF/ICSI. Several RCT have proposed gonadotropin starting dose algorithms or gonadotropin starting dose modifications in order to improve live birth rates or to reduce treatment risks. On their own, these RCTs are less generalizable and smaller than an individual participant data (IPD) meta-analysis (IPD-MA) and most of them only look at either live birth rates or safety instead of combining both outcomes. Therefore aim of the present study was to develop and validate a gonadotropin starting dose-selection model using all available data on gonadotropin-alpha and -beta dosing in IVF/ICSI from existing RCTs, to predict both the chance of live birth and treatment risk per gonadotropin starting dose. Clinicians could use these predictions to choose an individualized gonadotropin starting dose.

## Methods

### Data acquisition

IPD was collected by the ORT iOS IPD-MA study group with two aims (PROSPERO registration CRD42019115489): (i) to compare individualized dosing to standard dosing (this will be a different scientific publication) and (ii) to develop and validate a gonadotropin dose selection model (the current publication). This study was conducted according to the PRISMA-IPD statement and the PICOTS Framework (Samson and Schoelles, 2012; Stewart *et al.*, 2015). A systematic search was undertaken for (un)published RCTs comparing different gonadotropin starting doses in women undergoing IVF/ICSI, where dose selection was based on at least one type of ORT and live birth or ongoing pregnancy was reported. The Cochrane Gynaecology and Fertility Group Specialised Register, Cochrane Central Register of Studies Online, MEDLINE, Embase, CINAHL, LILACS, DARE, ISI Web of Knowledge, ClinicalTrials.gov, OpenGrey, and the World Health Organisation International Trials Registry Platform search portal were searched. For further details, please refer to Supplementary Files S1 and S2. The search strategy was based on that in the Cochrane systematic review of Lensen *et al.* (2024) the last search was performed on 13 July 2022. Details can be found in Supplementary Files S1 and S2. Similar to the systematic review conducted by Lensen *et al.* (2024), we have opted to exclude non-RCTs due to the higher risk of bias (RoB) (Higgins *et al.*, 2011). Moreover, randomization allocates patients to specific dosing groups. This ensures that the observed differences in the outcomes are most likely due to the FSH dosing choice, and much less created by confounding factors. An important aim was to develop a generally applicable prediction tool to use whenever a clinician has a new patient in front of them, for whom they do not yet have any information regarding their ovarian response status. To serve all patients with this dosing tool, it is necessary to use study populations with low, normal, and high ovarian responders. This will help clinicians managing any patient indicated to start IVF/ICSI.

Screening on title/abstract was performed by two independent reviewers (R.W. and N.S.) using Covidence. All eligible studies were assessed on full text for eligibility independently by the same reviewers. Authors were contacted in case of doubts. Disagreements were resolved by discussion with a third and fourth reviewer (F.B. and H.T.). Corresponding authors of eligible studies were invited to join the ORT iOS IPD-MA study group. After signing a data transfer agreement, their IPD was transferred through a secured transfer system. Institutional Review Board on ethics approval was confirmed.

### Data preparation

In total, 14 databases were transformed into a uniform format and variables were standardized (for additional details, see Supplementary File S3). Data cleaning included checking inclusion and exclusion criteria, duplicates, structural errors, missing data, outliers and performing consistency checks (for instance comparing averages or numbers reported in paper to dataset, checking impossible values or combinations). When these checks raised any issues with respect to data integrity, we tried to resolve this with the corresponding authors; studies were excluded if the issues could not be resolved.

IPD sheets included the following candidate predictors for the prediction model: female age, body mass index (BMI), anti-Müllerian hormone (AMH), antral follicle count (AFC), basal follicle-stimulating hormone (bFSH), gonadotropin starting dose (GSD), duration of subfertility, cycle length, gonadotropin-releasing hormone (GnRH) analog (agonist/antagonist), insemination method (IVF/ICSI), previous IVF/ICSI cycles, type of subfertility (primary/secondary), gravidity, parity and cause of subfertility. In addition, outcome data were received, such as dose adjustments, total number of days of gonadotropin stimulation, total gonadotropin dose, cancellation due to hyper-response, cancellation due to low response, underwent oocyte retrieval (yes/no), number of oocytes, number of fresh embryos, fresh transfer (yes/no), number of fresh embryos transferred, total number of embryos, number of cryopreserved embryos, clinical pregnancy (yes/no), ongoing pregnancy (yes/no), live birth (yes/no), development of moderate or severe OHSS (yes/no), freezing all embryos due to OHSS risk (yes/no), coasting during ovarian stimulation (yes/no). Combined treatment risk was defined as the development of moderate or severe OHSS and/or any measure taken to prevent the development of OHSS, including cycle cancellation due to an excessive response, coasting, GnRH agonist triggering, freezing all embryos and/or no embryo transfer to prevent the development of OHSS. As the included studies did not use the GnRH agonist trigger to reduce the incidence of OHSS, this was not included and therefore the intent of the initiated IVF/ICSI cycle was to perform a fresh embryo transfer. For other definitions and units of the variables, see Supplementary File S3.

Missing data were noted in some of the datasets, in spite of many efforts to stimulate the use of a core set of variables in prospective studies (Wilkinson *et al.*, 2016). Multi-level multiple imputation offers a powerful tool for handling missing data and harmonizing variable sets in meta-analyses where studies exhibit variations in reported variables. Therefore, it was decided to include datasets that (i) had at least ongoing pregnancy or live birth as an outcome measure, (ii) reported information about the risks of ovarian hyperstimulation, and (iii) reported at least one ORT. The aforementioned imputation method was then used to handle missing data.

### Risk of bias

RoB was already assessed for the Lensen *et al.* (2024) Cochrane systematic review except for two studies (Youssef *et al.*, 2017; Friis Petersen *et al.*, 2019). One reviewer (N.S.) independently assessed all included studies again using the Cochrane RoB assessment tool, and included the two additional studies (see Supplementary File S4) (Higgins *et al.*, 2011).

### Statistical analysis

Missing data were imputed 100 times with 20 between-imputation iterations (for more details, see Supplementary File S5).

#### Variable selection stage

First, internal–external cross-validation (IECV) sets were made to select predictors. To fit a model, all studies except for two were selected (repeated 14 times in different combinations, so every study was excluded twice) and to test the resulting model, the first study that was not included in the fitting stage was selected. Backwards selection was performed on all candidate predictors per imputation set, per IECV set with generalized linear mixed models using the ‘psfmi’ package in R. We assessed model discrimination (using concordance-statistics) and pooled results using Rubin’s Rules. Per backwards selection step, model performance was assessed and the least predictive variable was excluded, until the most predictive variables were left.

#### Model development stage

Different combinations with the most predictive variables from the variable selection stage were used in the model development stage. The 13 studies from the variable selection stage (12 studies to fit the model + 1 study to test the model) were used to fit the models and the one study that had not been used yet was selected to validate the models. Only models in studies with at least one event were validated. Forest plots of the performance measures were used and summary statistics were calculated using a random effects meta-analysis estimate. This resulted in a summary area under the curve (AUC), summary calibration slope and summary calibration-in-the-large (CIL) per model.

An optimal model with the highest performance was developed. A model that is easy to adapt into routine patient care (with less variables that are easy to obtain) and with the highest performance was developed as the ‘clinically feasible’ model. The final models (the optimal and clinically feasible models for both live birth and combined treatment risk) were developed using a one-stage approach with all available data pooled, using accounting for clustering within studies.

For details on the IECV, variable selection and model development, see Supplementary File S6.

## Results

### Study selection

**Figure 1.**
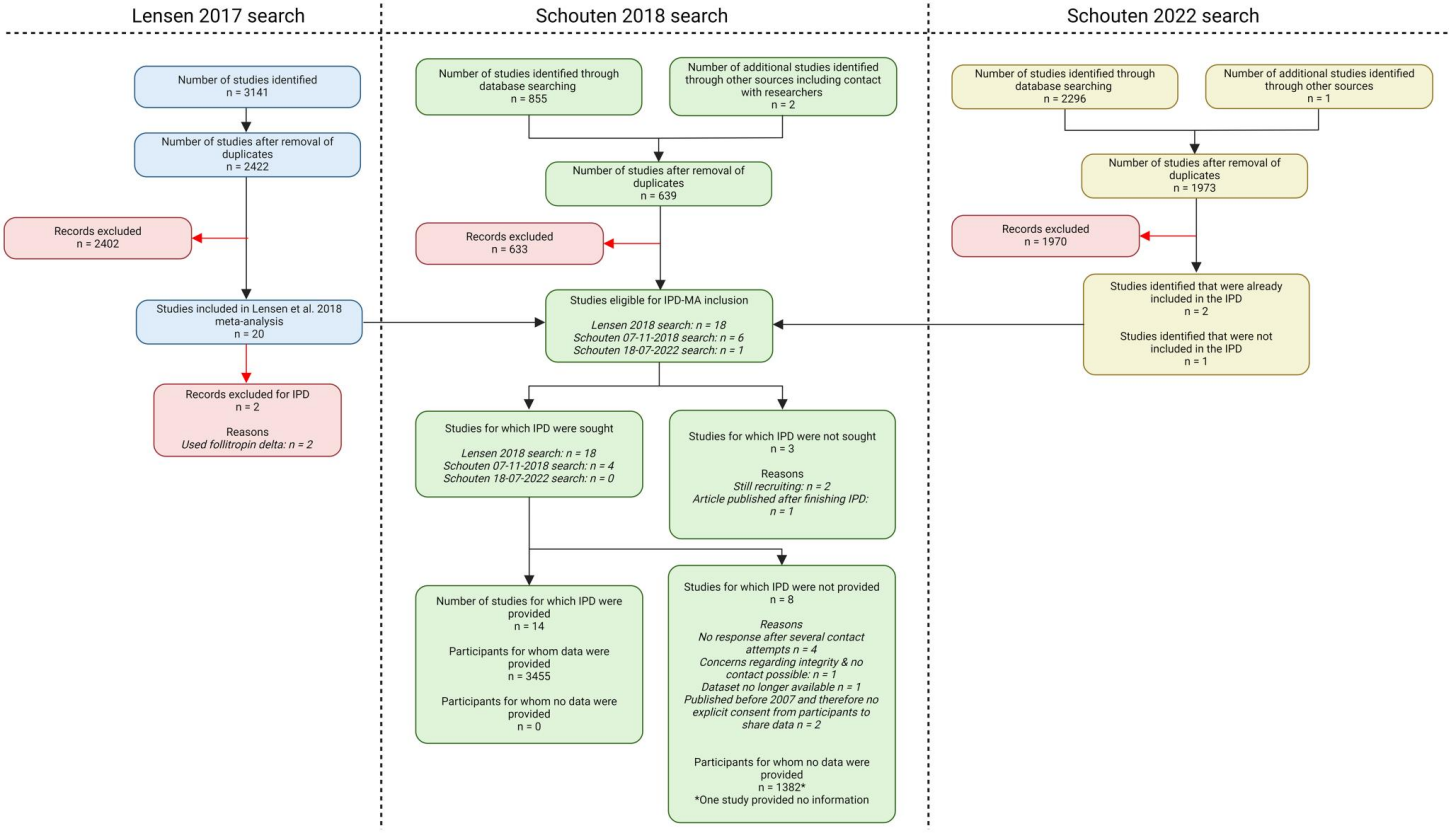
PRISMA flow diagram for individual patient data systematic reviews.

In total 639 articles were screened based on title/abstract, assessed on full text for eligibility, and initially, 24 studies were eligible for inclusion. Of the total 24 studies, IPD was sought for 22 of them and 15 provided their IPD for in total 3933 participants. Of the 15 studies, one was not included in the current analysis after data transfer due to questions regarding data inconsistencies that were not answered before the data analysis started (Youssef *et al.*). The last search resulted in the identification of one study, IPD was not sought due to the timeline of the analysis of our study (Liu *et al.*, 2022). The process of study selection and inclusion has been displayed in Fig. 1.

### Study characteristics

After the exclusion of women who fell outside of the scope of this IPD, data of 3455 women remained. After extensive modeling, cases with female age of 39 years and over were excluded. In these women, the incidence of OHSS appeared very low and treatment risk events may be considered very rare events. These cases disrupted the effect of the interaction between age and starting dose on combined treatment risk events but also on live birth. This exclusion step resulted in the final inclusion of 2907 women. The studies were performed in multiple countries and results were published between 2003 and 2019 (see Table 1). Gonadotropin starting dose ranged between 75 and 600 IU, and both GnRH agonist and antagonist studies were included. In one study, participants used a combination of human menopausal gonadotropin (hMG) and recombinant FSH (Bastu *et al.*, 2016) and in another study patients used a combination of hMG and urinary FSH (Lefebvre *et al.*, 2015). All other studies used recombinant FSH.

**Table 1.** Characteristics of included studies.

Study	Country	Number of centers	Randomized/eligible	Eligible <39 years	Type of responder^k^	Dose selection	Treatment	Gonadotropin range	Number of cycles	Fresh results	Frozen results	Fresh pregnancy outcome	Treatment risk report	Live birth	Combined treatment risk
Allegra *et al.* (2017) (published)	Italy	Single center	191/191	161	All	Nomogram (age, AMH, FSH) or standard (age ≤ 35 vs >35 years)	GnRH agonist + rFSH	125-225 IU	One cycle	Yes	No	Clinical pregnancy^a^	All	30.1%	12.4%
Bastu *et al.* (2016) (published)	Turkey	Single center	95/62^b^	48	LOR	300 versus 450 IU	GnRH antagonist + 1:1 hMG: rFSH	300-450 IU	One cycle	Yes	No	Ongoing pregnancy^a^	All	9.9%	0.0%
Friis Petersen (2019) (published)	Denmark	Dual-center (Denmark)	221/187^c^	187	All	AMH based (>24, 12–24 or <12 pmol/l) versus 150 IU	GnRH antagonist + rFSH	100–150 IU	One cycle	Yes	Yes	Livebirth	All	29.9%	1.6%
Jayaprakasan (2012) (published)	United Kingdom (UK)	Single center	135/130^d^	130	NOR^2^	225 versus 300 IU	GnRH agonist + rFSH	225–300 IU	One cycle	Yes	No	Livebirth	All	36.2%	4.6%
Klinkert (2005) (published)	Netherlands	Single center	52/52	15	LOR	150 versus 300 IU	GnRH agonist + rFSH	150-300 IU	One cycle	Yes	No	Livebirth	All	0.0%	0.0%
Klinkert (unpublished)	Netherlands	Single center	87/86^e^	37	LOR	300 versus 450 IU	GnRH agonist + rFSH	300-450 IU	One cycle	Yes	No	Livebirth	All	16.0%	0.0%
Lan *et al.* (2013) (published)	Vietnam	Single center	348/348	314	All^f^	AMH versus AFC^8^-based algorithm	GnRH agonist + rFSH	150-375 IU	One cycle	Yes	No	Livebirth	All	29.1%	8.9%
Lefebvre *et al.* (2015) (published)	Canada	Single center	366/366	232	LOR	450 versus 600 IU	GnRH agonist + 1:1 hMG: uFSH	450-600 IU	One cycle	Yes	No	Livebirth	All	15.5%	0.4%
Magnusson *et al.* (2017) (published)	Sweden	Single center	308/308	302	All	Conventional (age, BMI, AFC) versus AMH (conventional + AMH)	GnRH agonist + rFSH	75-300 IU	One cycle	Yes	No^g^	Livebirth	All	29.1%	8.3%
Olivennes *et al.* (2015) (published)	France, UK, Netherlands, Italy, Switzerland	Multicenter (22 centers in 9 European countries, 1 center in Chile)	200/200	200	NOR	CONSORT(age, height, weight, FSH, AFC) versus150 IU	GnRH agonist + rFSH	112.5-225 IU	One cycle	Yes	No	Livebirth	All	32.0%	6.0%
Oudshoorn *et al.* (2017) (published)	Netherlands	Multicenter (25 centers in The Netherlands)	521/519^h^	480	HOR^3^	100 IU versus 150 IU	GnRH agonist or antagonist + rFSH or uFSH	100-150 IU	Multiple cycles	Yes	Yes	Livebirth	All	26.3%	9.6%
Popovic-Todorovic *et al.* (2003) (published)	Denmark	Dual-center (Denmark)	262/262	249	All^i^	Nomogram (AFC, Doppler score, total ovarian volume, age, smoking) versus 150 IU	GnRH agonist + rFSH	100-250 IU	One cycle	Yes	No	Ongoing pregnancy^a^	All, except coasting	29.3%	4.3%
Tasker *et al.* (2010) (unpublished)	UK	Not stated in abstract or dataset	…^j^/238	203	All	Standard (age, FSH, estradiol and presence of PCO) versus standard + AMH + AFC	GnRH agonist + rFSH	100-450 IU	One cycle	Yes	No	Livebirth	All	31.3%	4.5%
Van Tilborg *et al.* (2017) (published)	Netherlands	Multicenter (25 centers in The Netherlands)	511/511	349	LOR	150 versus 225 or 450 IU (based on AFC)	GnRH agonist or antagonist + rFSH or uFSH	150-450 IU	Multiple cycles	Yes	Yes	Livebirth	All	18.9%	0.6%

LOR, predicted low ovarian responder; NOR, predicted normal ovarian responder; HOR, predicted high ovarian responder; AMH, anti-Müllerian hormone; FSH, follicle-stimulating hormone; IU, international units; BMI, body mass index; AFC, antral follicle count; CONSORT, CONsistency in r-FSH Starting dOses for individualized tReatmenT; PCO, polycystic ovaries; GnRH, gonadotropin-releasing hormone. Combined treatment risk = the development of moderate or severe ovarian hyperstimulation syndrome (OHSS) and/or any measure taken to prevent the development of OHSS, including cycle cancellation due to an excessive response, coasting, GnRH agonist triggering, freeze all and/or no embryo transfer to prevent the development of OHSS.

a Study did not report live birth, which was imputed using clinical and/or ongoing pregnancy.

b Group 3 consisting of 33 women used 150 FSH in combination with letrozole and was therefore excluded.

c 34 women used corifollitropin, which cannot be compared to FSH IU and were therefore excluded.

d We only received data of 130 women. Authors informed us that this was the final analysis Excel sheet and the original database was not accessible anymore.

e One woman did not start stimulation and had no case report file, therefore there was no data to be used for the prediction model.

f Women with basal FSH > 12 were excluded, but women with low AMH or AFC were included. Therefore population classified as ‘all’.

g In original dataset one patient was pregnant after frozen embryo transfer. We classified this patient as ‘not’ pregnant.

h Two women have missing cycle data, which is mentioned in the original article. One of them would be excluded because of her age.

i Women with basal FSH >12 were excluded, but women categorized as predicted ‘low’ or ‘high’ responders were included. Therefore population classified as ‘all’.

j Only abstract published, mentioned that 286 women were included, but not how many are randomized. We only received data of 238 women.

k As defined by original study.

Except for three studies (Popovic‐Todorovic *et al.*, 2003; Bastu *et al.*, 2016; Allegra *et al.*, 2017), all had IPD on live birth and OHSS. Live birth rates ranged from 0% to ∼36% and combined treatment risk events ranged from 0% to ∼12%. The reader is referred to Table 1 for an overview of the included studies. For outcome and variable definitions, see Supplementary File S3.

### Baseline characteristics for missing and non-missing data

Baseline characteristics for continuous data are shown in Table 2. Baseline characteristics per continuous variable before and after imputation are shown in Table 2. Means and standard deviations did not differ before and after imputation for the continuous variables. For the categorical variables, pre- and post-imputation numbers are very much comparable, except for gravidity and parity (Table 3). Type of infertility, gravidity, and parity cannot be imputed separately as they limit each other’s values: a primary subfertile patient cannot have gravidity 1 and parity 5. Therefore, post-imputation transformations were necessary to prevent impossible values, which resulted in differences between pre- and post-imputation gravidity and parity results.

**Table 2.** Baseline characteristics of dataset before and after imputation of the continuous variables.

	Preimputation % of missing data (n = 2907)	Preimputation mean	Preimputation SD	Postimputation mean	Postimputation SD
Age in years	0.0%	32.5	3.76	32.5	3.76
BMI in kg/m^2^	1.4%	23.3	3.65	23.3	3.66
Antral follicle count (2–10 mm)	1.5%	15.4	8.68	15.3	8.66
AMH in ng/ml	17.2%	3.3	2.67	3.2	2.66
bFSH in IU/l	25.6%	7.3	3.01	7.3	3.03
Starting dose in IU	0.0%	210.4	124.56	210.4	124.56
Duration subfertility in months	16.4%	37.7	25.56	38.6	26.96
Cycle length in days	37.4%	28.3	2.04	28.2	2.30
Number of stimulation days	1.3%	11.0	2.43	11.0	2.42
Total gonadotropin dose in IU	1.5%	2371.2	1556.01	2380.6	1570.33
Number of oocytes	0.0%	8.9	6.29	8.9	6.29
Number of usable embryos	1.9%	2.8	2.80	2.8	2.78
Number of cryopreserved embryos	1.9%	1.5	2.52	1.5	2.50

On the left side of the table the percentage of missing data per variable before imputation is presented, with the corresponding pre-imputation mean and standard deviation (SD). On the right side of the table, the post-imputation results are presented. As missing data was imputed 100 times, there are 100 times as many observations. Pre- and post-imputation results are comparable.

AMH, anti-Müllerian hormone; FSH, follicle-stimulating hormone; IU, international units; BMI, body mass index.

**Table 3.** Baseline characteristics of dataset before and after imputation of the categorical variables.

Variable	% missing data	Label	Preimputation number (%) n = 2907	Postimputation number (%) n = 2907
GnRH-protocol	0.0%	Antagonist	426 (14.7)	426 (14.7)
		Agonist	2481 (85.3)	2481 (85.3)
IVF	3.1%	No	1533 (54.4)	1580 (54.4)
		Yes	1285 (45.6)	1327 (44.9)
ICSI	3.1%	No	1279 (45.4)	1306 (44.9)
		Yes	1539 (54.6)	1601 (55.1)
Previous ART cycles	2.2%	No	2520 (88.6)	2571 (88.5)
		Yes	324 (11.4)	336 (11.5)
Type of infertility	21.5%	Primary	1421 (62.3)	1801 (62.0)
		Secondary	861 (37.7)	1106 (38.0)
Gravidity	22.7%	0	1421 (63.3)	1801 (62.0)
		1	567 (25.2)	568 (19.5)
		2	62 (2.8)	62 (2.1)
		3	14 (0.6)	14 (0.5)
		4	7 (0.3)	7 (0.2)
		5	2 (0.1)	2 (0.1)
		6	1 (0.0)	1 (0.0)
		7	1 (0.0)	1 (0.0)
		8	1 (0.0)	1 (0.0)
		9	1 (0.0)	1 (0.0)
		10	1 (0.0)	1 (0.0)
Parity	26.9%	0	1833 (86.2)	2213 (76.1)
		1	240 (11.3)	362 (12.4)
		2	44 (2.1)	323 (11.1)
		3	8 (0.4)	8 (0.3)
		5	1 (0.0)	1 (0.0)
Cause of infertility	1.7%	Unknown	893 (31.3)	909 (31.3)
		Female	641 (22.4)	652 (22.4)
		Male	1231 (43.1)	1252 (43.1)
		Mixed	55 (1.9)	56 (1.9)
		Other	37 (1.3)	38 (1.3)
Smoking	34.3%	No	1609 (84.3)	2405 (82.7)
		Yes	300 (15.7)	502 (17.3)
Dose adjustments	0.6%	No	2276 (78.8)	2290 (78.7)
		Yes	613 (21.2)	617 (21.2)
Cancel hyper response	0.0%	No	2849 (98.0)	2849 (98.0)
		Yes	58 (2.0)	58 (2.0)
Cancel poor response	0.0%	No	2694 (92.7)	2694 (92.7)
		Yes	213 (7.3)	213 (7.3)
Follicle puncture	0.0%	No	304 (10.5)	304 (10.5)
		Yes	2603 (89.5)	2603 (89.5)
Fresh embryo transfer	0.0%	No	604 (20.8)	604 (20.8)
		Yes	2303 (79.2)	2303 (79.2)
Number of fresh embryos transferred	0.0%	0	604 (20.8)	604 (20.8)
		1	1348 (46.4)	1348 (46.4)
		2	695 (23.9)	695 (23.9)
		3	87 (3.0)	87 (3.0)
		4	172 (5.9)	172 (5.9)
		5	1 (0.0)	1 (0.0)
Clinical pregnancy	0.8%	No	1967(68.2)	1983 (68.2)
		Yes	916 (31.8)	924 (31.8)
Ongoing pregnancy	6.9%	No	1967 (72.7)	2089 (71.9)
		Yes	738 (27.3)	818 (28.1)
Live birth	16.6%	No	1803 (74.4)	2137 (73.5)
		Yes	620 (25.6)	770 (26.5)
Freeze all	0.0%	No	2860 (98.4)	2860 (98.4)
		Yes	47 (1.6)	47 (1.6)
Coasting	8.9%	No	2632 (99.4)	2889 (99.4)
		Yes	15 (0.6)	18 (0.6)
Moderate or severe OHSS	6.2%	No	2675 (98.1)	2849 (98.0)
		Yes	53 (1.9)	58 (2.0)
Combined safety	6.1%	No	2573 (94.3)	2744 (94.4)
		Yes	156 (5.7)	163 (5.6)

On the left side of the table, the percentage of missing data per variable before imputation is presented, with the corresponding pre-imputation number of events (and percentage) and post-imputation number of events (and percentage). Percentage of missing data post-imputation are not shown, all are 0% missing. Pre- and post-imputation results are comparable, except for parity and gravidity.

### Missing data

In Supplementary File S5, the convergence plots are visualized. Both baseline tables and the convergence plots confirm adequate imputation of missing variables.

### IPD integrity and risk of bias within studies

Almost all studies (n = 12, 86%) had high RoB for blinding participants and personnel and outcome assessment (for OHSS), since both participants and personnel were aware which gonadotropin dose was used. As can be seen in Table 4, 2 of 14 studies have an overall high RoB and their results should be interpreted carefully (Klinkert *et al.*, unpublished; Tasker *et al.*, 2010). For one study we labeled blinding as unclear, as the abstract does not mention anything on blinding (Tasker *et al.*, 2010). For the same study, we classified other biases as high risk because only an abstract was available.

**Table 4.** Overview of risk of bias assessment.

		Sequence generation	Allocation concealment	Blinding participants personnel	Blinding outcome assessment	Incomplete outcome data	Selective outcome reporting	Other bias
1	Allegra *et al.* (2017)	Low	Low	High	High	Low	High	High
2	Bastu *et al.* (2016)	Low	Low	Unclear	Unclear	Low	Low	Low
3	Friis Petersen (2019)	Low	Low	Low	Low	Low	Low	Low
4	Jayaprakasan *et al.* (2010)	Low	Low	High	High	Low	Low	Low
5	Klinkert (unpublished)	Low	Low	High	High	High	High	High
6	Klinkert (2005)	Low	Low	High	High	Low	High	Low
7	Lan *et al.* (2013)	Low	Low	High	High	Low	High	Low
8	Lefebvre *et al.* (2015)	Low	Low	High	High	Low	Low	Low
9	Magnusson *et al.* (2017)	Low	Low	Low	Low	Low	Low	Low
10	Olivennes *et al.* (2015)	Low	Low	High	High	Low	Low	Low
11	Oudshoorn *et al.* (2017)	Low	Low	High	High	Low	Low	Low
12	Popovic-Todorovic *et al.* (2003)	Low	Low	High	High	Low	High	Low
13	Tasker *et al.* (2010)	Low	Low	High	Unclear*	High	High	High*
14	Van Tilborg *et al.* (2017)	Low	Low	High	High	Low	Low	Low

Risk of bias classified as low, unclear or high risk of bias. The asterisk symbol indicates disagreement with risk of bias label in the previously mentioned meta-analysis (Lensen *et al.*, 2024). For one study (Tasker 2010), we scored blinding outcome as unclear instead of low and other bias as high instead of unclear (FDA, 2019).

The integrity check of the Tasker *et al.* (2010) was difficult to assess since this study only published an abstract with little information (Supplementary File S7). As the data that was available passed the consistency checks, we decided not to exclude this study, but to interpret the results with caution. Three studies were not registered in trial registries (Klinkert *et al.*, 2005; Tasker *et al.*, 2010; Klinkert *et al.*, unpublished), but because both studies were completed before the publication of the Section 801 of the Food and Drug Administration Amendments Act of 2007, inclusion was deemed to be acceptable (FDA, 2019).

### Predictor selection stage

Models were tested with splines with three (and more) knots on age, BMI, and starting dose. Age and starting dose were centered around the mean to assure a relevant interpretation of the intercept. Starting dose was scaled (divided by 10) and forced into the model. An interaction between age and starting dose was modeled using centering of age and dose around their study specific means as recommended by Riley to separate within and between study effects (Riley *et al.*, 2020).

For predictor selection, there were 19 exclusion steps resulting in 19 live birth models per set, for combined treatment risk there were 18 (since AMH was never excluded due to high significance). As one study did not contain any live birth or safety events, this study could not be used to validate both models (Klinkert *et al.*, 2005). Two studies did not have any combined treatment risk event and therefore could not be used to validate the model (Bastu *et al.*, 2016; Klinkert *et al.*, unpublished).

### Model development and validation

#### Internal–external cross-validation optimal model live birth

Forest plots of the performance measures were used and summary statistics were calculated using a random effects meta-analysis estimate, resulting in a summary AUC, summary calibration slope and summary CIL for each model (see figures in Supplementary File S8).

**Figure 2.**
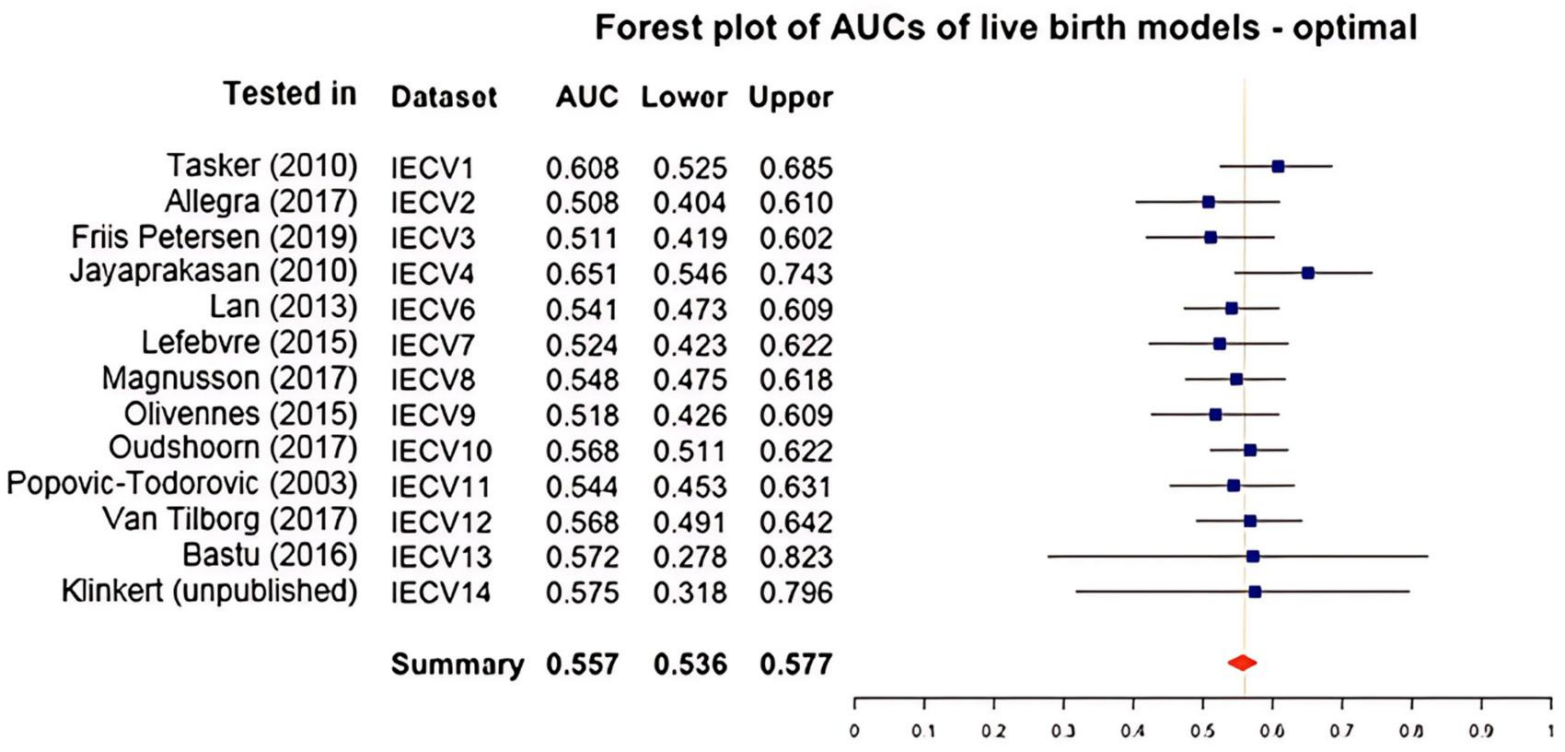
**Forest plot of AUCs of the optimal live birth prediction models including age, starting dose, body mass index, AFC, fertilization with IVF or ICSI, and AMH.** IECV, internal external cross-validation set; Lower, lower 95% of confidence interval; AUC, Area under the curve; Upper, upper 95% of confidence interval. Summary statistic results in an overall AUC of 0.557 (0.536–0.577) which is classified as poor to discriminate between women with and without a live birth.

The selected optimal prediction model for live birth included the predictors age, starting dose, BMI, AFC, IVF/ICSI, and AMH. This model used a spline on age with 3 knots and a log transformation of AMH. Figure 2 displays a forest plot with AUC per IECV set for the live birth model. The IECV summary statistic resulted in an overall AUC of 0.557 (95% confidence interval from 0.536 to 0.577). The overall IECV calibration slope of 0.410 (95% confidence interval from 0.012 to 0.808) is low: a perfect slope should be 1. This can be interpreted as an over-estimation of the results, meaning that the predictions are too extreme (Van Calster *et al.*, 2019). The overall IECV CIL of 0.065 (95% confidence interval from −0.123 to 0.252) is close to the perfect CIL of 0.

The AUCs for IECV set 1 (tested in Tasker *et al.* (2010)) and 4 (tested in Jayaprakasan *et al.* (2010)) were higher compared to the other sets. Both were large studies with a higher number of live birth events compared to the other studies. Results from set 1 (tested in Tasker *et al.* (2010)) should be interpreted with caution due to high RoB. The confidence intervals of IECV sets 13 (tested in Bastu *et al.* (2016)) and 14 (tested in Klinkert (unpublished)) were both larger than the other sets; both were very small studies with very low number of live birth events. Overall, validation of the model in different sets was stable and the prediction of live birth appeared poor, with the predictions at the extremes seeming to be overestimated.

#### Clinically feasible model: live birth

**Figure 3.**
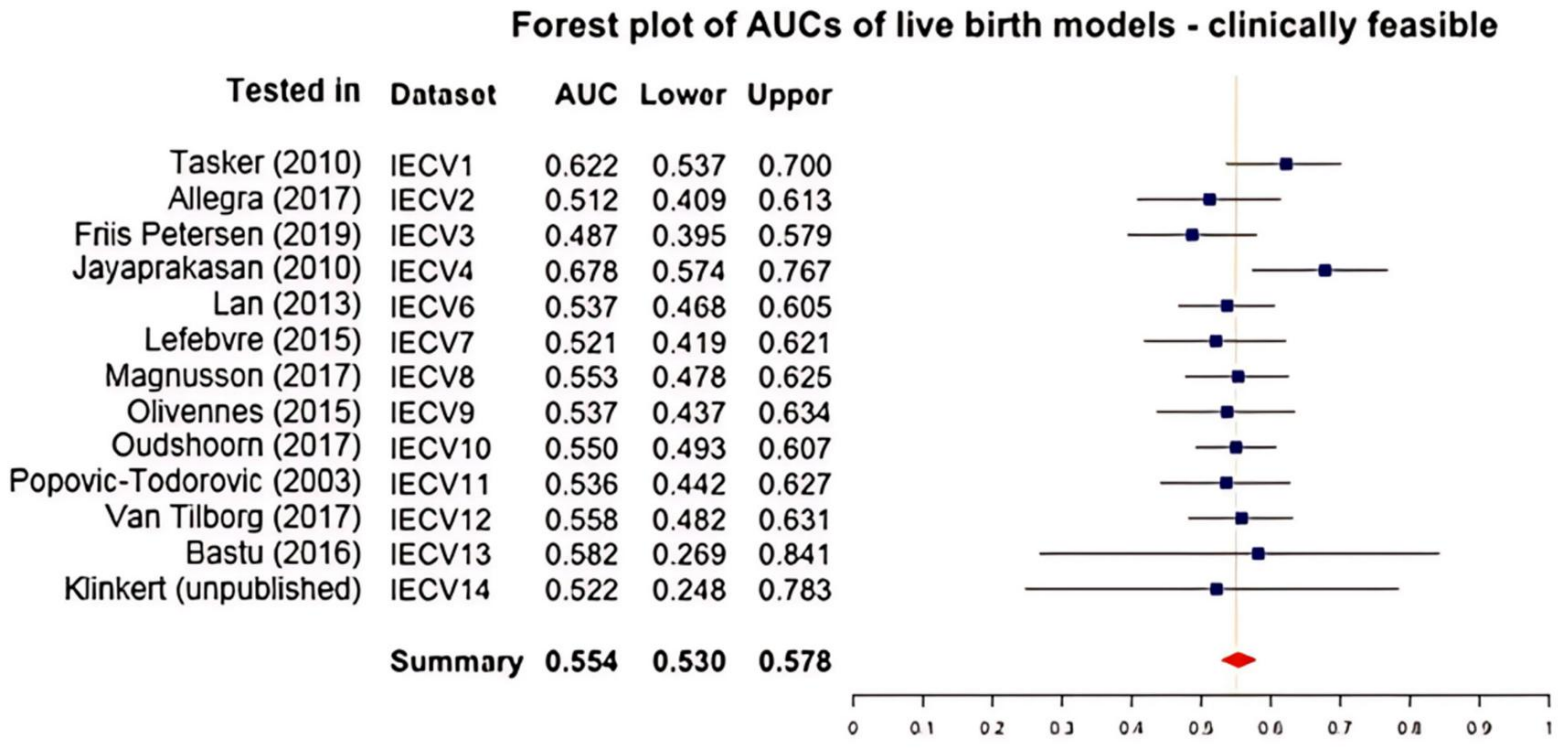
**Forest plot of AUCs of the clinically feasible live birth prediction models including age, starting dose, and AMH.** IECV, internal external cross-validation set; Lower, lower 95% of confidence interval; AUC, Area under the curve, Upper, upper 95% of confidence interval. Summary statistic results in an overall AUC of 0.554 (0.530–0.578) which is classified as poor to discriminate between women with and without a live birth.

Predictors included age (spline with three knots), starting dose and AMH (log transformed). As can be seen in Fig. 3, the IECV summary statistic resulted in an overall AUC of 0.554 (95% confidence interval from 0.530 to 0.578) and overall IECV calibration slope of 0.506 (95% confidence interval from 0.069 to 0.943). The overall IECV CIL of 0.064 (95% confidence interval from −0.125 to 0.254) was close to the perfect CIL of 0.

The AUCs for IECV set 1 (tested in Tasker *et al.* (2010)) and 4 (tested in Jayaprakasan *et al.* (2010)) were again higher than the other sets. Also, the confidence intervals of IECV sets 13 (tested in Bastu *et al.* (2016)) and 14 (tested in Klinkert (unpublished)) were both larger than the other sets. As compared to the optimal model, this model has a comparable discriminative performance, with a slightly better calibration.

#### Internal–external cross-validation optimal model combined treatment risk

Two models were selected as the optimal model for combined treatment risk, as their performance was equal.

##### Optimal model 1: combined treatment risk

**Figure 4.**
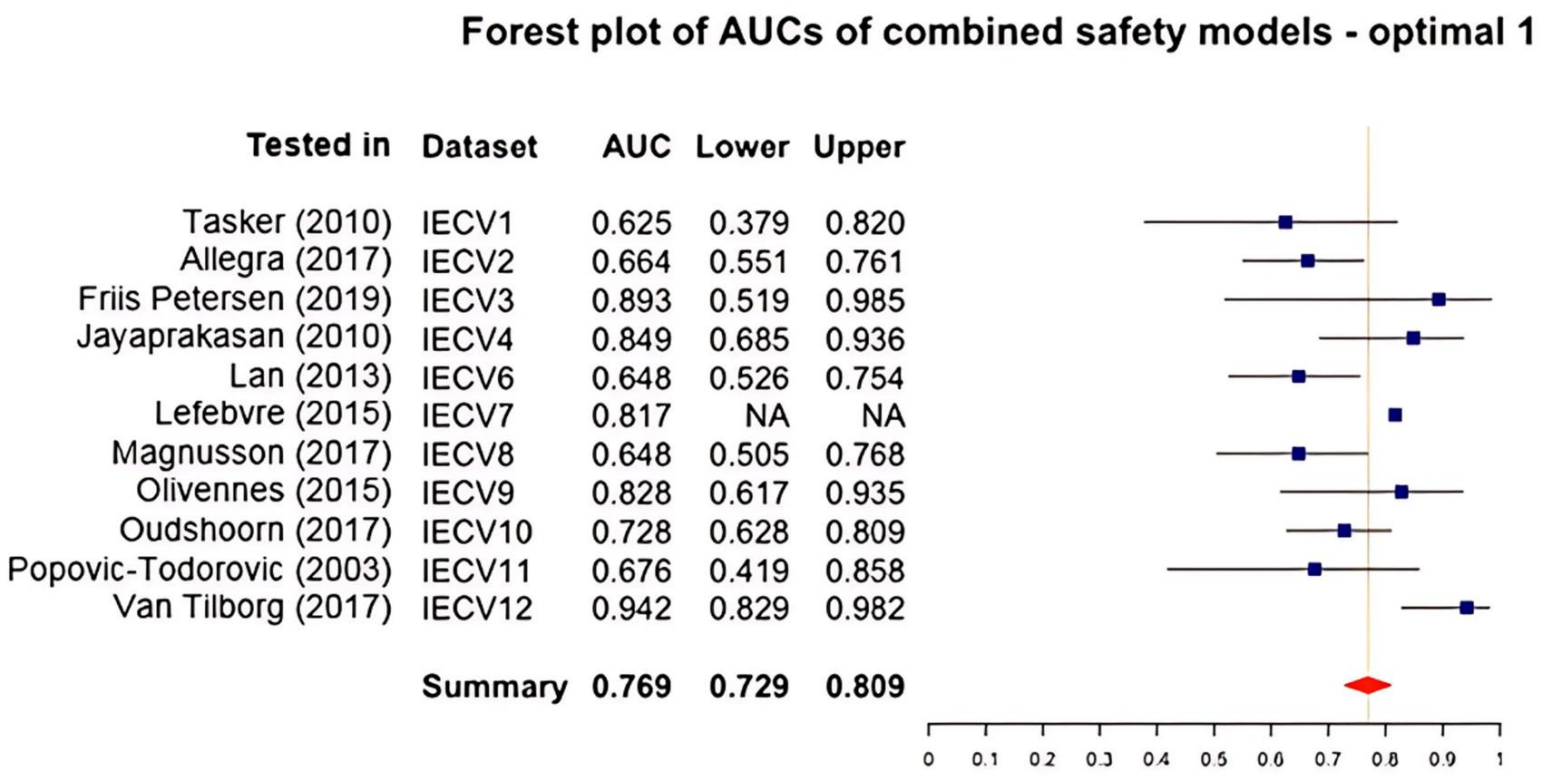
**Forest plot of AUCs of the first optimal combined treatment risk prediction models including age, starting dose, AMH and basal follicle-stimulating hormone.** IECV, internal external cross-validation set; Lower, lower 95% of confidence interval; AUC, area under the curve; Upper, upper 95% of confidence interval. Summary statistic results in an overall AUC of 0.769 (0.729–0.809) which is classified as acceptable to discriminate between women with and without a combined treatment risk event.

This model included the predictors age, starting dose (scaled), AMH and bFSH. Figure 4 displays the overall AUC of 0.769 (95% confidence interval from 0.729 to 0.809). The overall IECV calibration slope was 1.082 (95% confidence interval from 0.598 to 1.567). The overall IECV CIL was 0.223 (95% confidence interval from −0.412 to 0.858).

##### Optimal model 2: combined treatment risk

**Figure 5.**
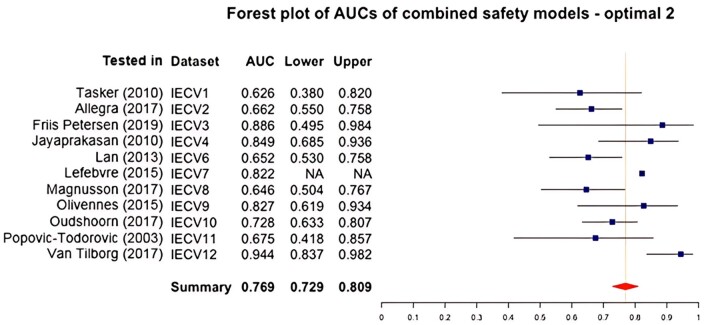
**Forest plot of areas under the curve (AUCs) of the second optimal combined treatment risk prediction models including age, starting dose, AMH, basal follicle-stimulating hormone, and GnRH analogue.** IECV, internal external cross-validation set; Lower, lower 95% of confidence interval; AUC, area under the curve; Upper, upper 95% of confidence interval. Summary statistic results in an overall AUC of 0.769 (0.729–0.809) which is classified as acceptable to discriminate between women with and without a combined treatment risk event.

This model included the predictors age, starting dose (scaled), AMH, bFSH, and GnRH analog. Figure 5 displays the overall AUC of 0.769 (95% confidence interval from 0.729 to 0.809). The overall IECV calibration slope was 0.987 (95% confidence interval from 0.553 to 1.421). The overall IECV CIL was 0.208 (95% confidence interval from −0.421 to 0.836).

In both models, AUCs that stand out were set 12 (higher compared to the rest; tested in Van Tilborg *et al.* (2017)) and set 3 (broader confidence interval compared to the rest; tested in Friis Petersen (2019)). Both studies had a low number of safety events and had a lower relative weight in calculating the summary statistic. The CIL was extremely high in set 7 (tested in Lefebvre *et al.* (2015)), indicating an underestimation of the prediction. However, there was only one event in this model testing set: a miss-prediction can have an enormous impact on the calibration plot.

Overall, the predictive accuracy of both optimal combined treatment risk models appeared to be sufficient (Hosmer *et al.*, 2013).

#### Clinically feasible model: combined treatment risk

**Figure 6.**
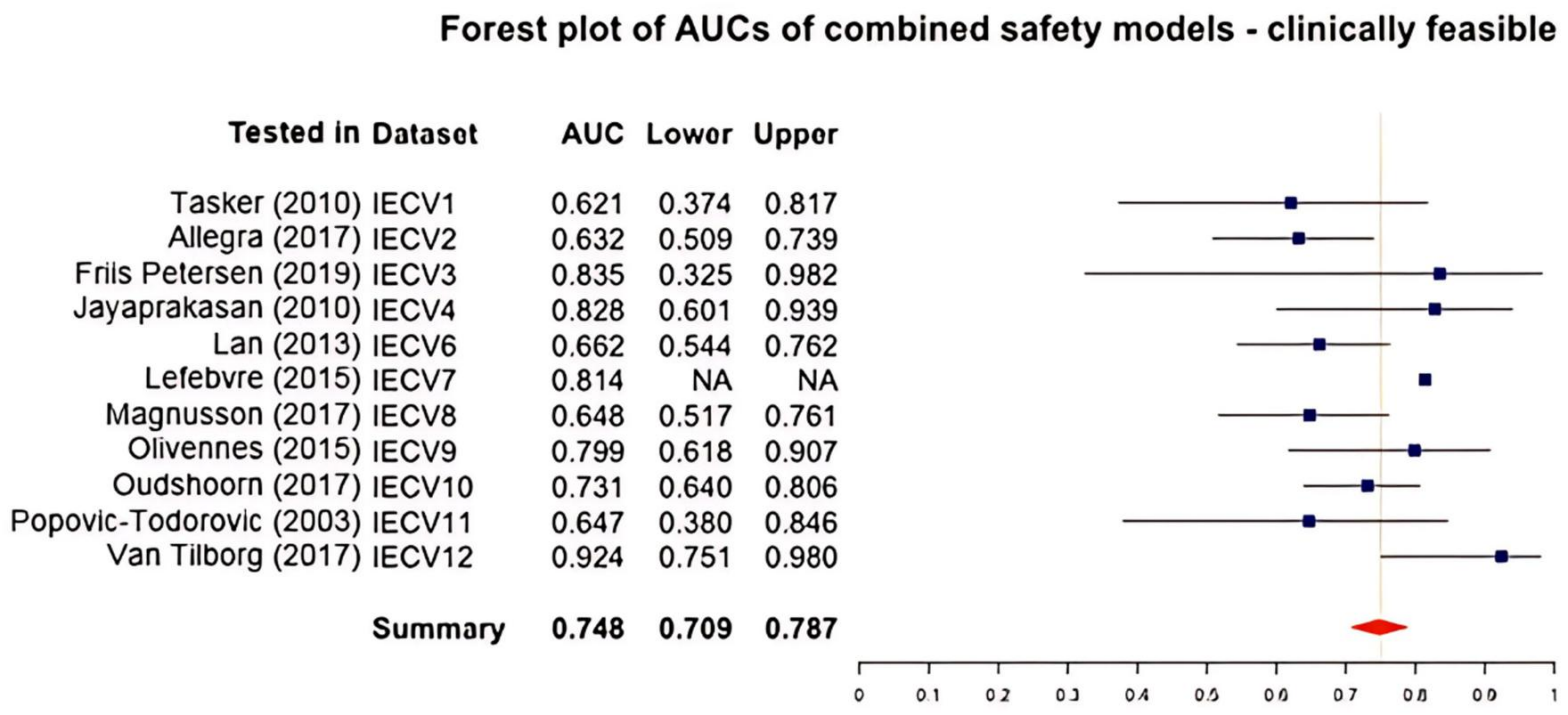
**Forest plot of areas under the curve (AUCs) of the clinically feasible combined treatment risk models including age, starting dose, AMH and GnRH analogue.** IECV, internal external cross-validation set; Lower, lower 95% of confidence interval; AUC, area under the curve; Upper, upper 95% of confidence interval. Summary statistic results in an overall AUC of 0.748 (0.709–0.787) which is classified as acceptable to discriminate between women with and without a combined treatment risk event.

The predictors age, starting dose (scaled), AMH, and GnRH analog were included. The overall AUC was 0.748 (95% confidence interval from 0.709 to 0.787), see Fig. 6. The overall IECV calibration slope was 1.019 (95% confidence interval from 0.563 to 1.476). The overall IECV CIL was 0.207 (95% confidence interval from −0.374 to 0.788).

Trends seen in the selected optimal models were more apparent in the clinically feasible safety model: the model had a higher performance (higher AUCs) in studies with lower incidences of safety events. IECV set 10 (tested in Oudshoorn *et al.* (2017)) was closest to the summary statistic and was also the study with the most combined treatment risk events. This was compatible with the calibration slope and CIL findings: there was an overestimation in set 1 (tested in Tasker *et al.* (2010)) and 12 (tested in Van Tilborg *et al.* (2017); with low combined treatment risk events). The slope of set 3 (tested in Friis Petersen (2019)) was extremely high and had a very large confidence interval, this was also due to a low number of events (n = 3) and should be interpreted with caution. In conclusion, this model functions well in discriminating between women with and without a combined treatment risk event. However, in a population that is at risk of having a combined treatment risk event, this model underestimates the risk of developing an event due to regression to the mean and vice versa for a population that has a low risk of having a combined treatment risk event. Predictions of the optimal models are more stable in different settings compared to the clinically feasible model, but the clinically feasible model is easier to implement in routine practice.

### Clinical applicability

Examples of the dose calculator as a clinically applicable tool and based on the model selections as outlined in the aforementioned can be seen in Tables 5 and 6. This model is not applicable for women who plan to have a freeze-all strategy, women who have PCOS, or women who use medication other than gonadotropin alpha, gonadotropin beta or hMG (see Supplementary File S1). It shows the treatment risk and the live birth chance per gonadotropin starting dose. It is recommended to choose the highest starting dose with an acceptable risk, to minimize the risk of a low response. If a treatment risk of 3% is deemed acceptable, it is advised to use 137.5 international units FSH in the first example.

**Table 5.** Example 1 of the FSH dose-calculator.

Dose (in IU)	Live birth chance	Safety risk
100.0	28%	2.9%
112.5	28%	3.0%
125.0	28%	3.0%
137.5	28%	3.0%
150.0	28%	3.1%
162.5	28%	3.1%
175.0	28%	3.2%
187.5	28%	3.2%
200.0	28%	3.2%
212.5	28%	3.3%
225.0	28%	3.3%

Example for a 34-year-old patient using a GnRH antagonist protocol with AMH level of 5.3 µg/l. As can be seen, this patient has an increased treatment risk with a higher gonadotropin starting dose. If a treatment risk of 3.0% is deemed acceptable in a specific clinic, this patient is advised to start with 137.5 IU FSH.

**Table 6.** Example 2 of the FSH dose-calculator.

Dose (in IU)	Live birth chance	Safety risk
100.0	28%	7.6%
112.5	28%	7.7%
125.0	28%	7.8%
137.5	28%	7.9%
150.0	28%	7.9%
162.5	28%	8.0%
175.0	28%	8.1%
187.5	28%	8.2%
200.0	28%	8.3%
212.5	28%	8.4%
225.0	28%	8.5%

Example for a 34-year-old patient using a GnRH agonist protocol with AMH level of 5.3 µg/l. As can be seen, this patient has an increased treatment risk with a higher gonadotropin starting dose. If a treatment risk of 3.0% is deemed acceptable in a specific clinic, this patient is advised to start with 100 IU FSH.

## Discussion

Using IPD from world-wide RCTs, a gonadotropin dose selection model was developed and validated. It allows for accurate discrimination of patients with higher and lower treatment risk. In contrast, the role of FSH dose selection in optimizing the probability of live birth appeared poor. This model may aid clinicians and patients in choosing an appropriate (FSH) starting dose and in counseling about the treatment risks, based on robust, objective parameters instead of a physician’s subjective interpretation or valuation of patient characteristics.

The poor utility of dose selection for live birth rates may not be a surprise as in the last 20 years, 35 prediction models have been presented with the aim of predicting live birth rates (Ratna *et al.*, 2020). Almost all had an AUC < 0.7, which is classified as poorly capable of discriminating between patients with and without live birth, emphasizing that predicting live birth remains a difficult task (Hosmer *et al.* (2013)). Finding that gonadotropin starting dose does not have an impact on live birth prospects for the couple is in line with a recent extensive meta-analysis that included all gonadotropin dosing studies up until 2018, revealing that altering the gonadotropin starting dose does not have any effect on live birth rates after a fresh embryo transfer (Lensen *et al.*, 2024). It is therefore important to switch focus towards the role of gonadotropin dosing in managing treatment risks instead of live birth.

There are several strategies to apply in IVF/ICSI treatment to manage safety and become an OHSS-free clinic. Specifically the GnRH agonist triggering is considered an effective and popular strategy for patients with a hyper-response (TEGG Ovarian Stimulation *et al.*, 2020). However, when planning to perform a fresh embryo transfer, it is relevant to develop and validate a gonadotropin starting dose selection model as some centers may still prefer a GnRH agonist downregulation protocol for better ART treatment planning and scheduling. Moreover, 2.0–5.5% of patients have an insufficient LH response to GnRH agonist trigger (Chen *et al*., 2012; Kummer *et al.*, 2013; Meyer *et al.*, 2015; Chang *et al*., 2016; Lu *et al.*, 2016). A suboptimal response in terms of the oocyte recovery rate per follicle may occur due to an insufficient LH response caused by irregularities in the biological activity of the GnRH agonist, or in patients with a hypothalamic-pituitary dysfunction, a low BMI, GnRH receptor mutations or patients who have used oral contraceptives for a long period (Zegers-Hochschild *et al.*, 1995; Chevrier *et al.*, 2011; Kummer *et al.*, 2013; Meyer *et al.*, 2015). On the other hand, an insufficient oocyte output also occurs after hCG trigger (Revelli *et al.*, 2017). Two studies show comparable empty follicle syndrome rates, however, these studies included healthy oocyte donors, which is not a good representation of the IVF/ICSI population (Castillo *et al.*, 2012; Christopoulos *et al.*, 2015). More studies are required to draw strong conclusions regarding oocyte output after GnRH agonist or hCG triggering.

Even with the agonist trigger approach and the freeze-all protocol, a normal instead of an excessive response may still be preferable from other perspectives. The recruitment of a large cohort of follicles causes more patient discomfort, creates more difficult oocyte retrieval procedures, and may increase the risk of early OHSS manifestations and thromboembolic events or bleeding after an intensive follicle aspiration procedure or the risk of adnexal torsion due to enlarged ovaries (Bodri *et al.*, 2008; Magnusson *et al.*, 2018; Mizrachi *et al.*, 2020). From this point of view, the trend is towards targeting to retrieve ∼15–20 oocytes even in freeze-all cycles, to optimally balance safety and efficacy (Mizrachi *et al.*, 2020). Optimizing the patients’ wellbeing starts with choosing a personalized and optimal gonadotropin starting dose (Devroey *et al.*, 2011).

### Strengths and limitations

This is the largest collaboration on dose prediction and the first to combine safety, live birth, and dose selection in one prediction model for various FSH preparations. We used live birth instead of the more commonly used clinical pregnancy and we studied all measures to prevent OHSS and the development of moderate and/or severe OHSS. This study combined 14 different datasets from RCTs, with many potential predictors, which could be used to internally-externally develop and validate this easy-to-use prediction tool. This model is validated in different geographical and temporal situations, and we provided the possibility to adjust the dose selection model to a clinic’s characteristics (mean age and mean starting dose).

Despite the strengths, this study also has limitations. As raw data are imperative for a prediction model, we could not use information of studies where the authors were not willing or able to share their data. Therefore, there is a risk of reporting bias. However, the studies which we could not include were older than the included studies and did not use AMH or AFC as an ORT. As we used an intention-to-treat analysis, an underestimation of the outcome events may have occurred. The differences between studies were substantial, however, that is why age and starting dose were centered around the mean. The number of combined treatment risk events was low, especially in our IECV sets which resulted in less stable validation results. This is of course due to the low incidence of combined treatment risk events in general and is therefore a challenge for all study groups. One way to handle small outcome numbers is to establish a collaboration of several study groups, which resulted in this current IPD-MA. Since we used existing data from RCTs, we were dependent on the study design and information collected by the original studies. We unfortunately did not have enough frozen cycle or multiple cycle results to include in this study. Therefore, we cannot exclude the possibility that women with a standard instead of lower gonadotropin starting dose have more frozen embryos and therefore a higher chance of having a second live born from one stimulation cycle (Schouten *et al.*, 2022). Moreover, we did not have substantial data on the agonist trigger and freeze-all policy or sufficient data to determine the influence of urinary versus recombinant FSH products, as the large majority of women used rFSH only.

Live birth was chosen as one of the main outcomes instead of the important intermediate variable number of oocytes, as it is the most clinically meaningful outcome, especially from the patient’s perspective. It directly represents the aim of the IVF and ICSI treatment: creating a pregnancy leading to the birth of a baby. Cumulative live birth rate was unfortunately not provided by many included studies and could therefore not be used as main outcome measure. As the direct result of dosing strategies will be differences in oocyte number yielded, one may argue that this variable could also have been used as outcome measure. Although oocyte number certainly will be an important factor in safety management, the role in live birth rates is much more puzzling. Retrospective cohort studies have shown that more oocytes are associated with a higher probability of live birth per fresh embryo transfer (up to 20 oocytes) and with a higher probability of cumulative live birth (up to 20–25 oocytes) (Sunkara *et al.*, 2011; Steward *et al.*, 2014; Vaughan *et al.*, 2017; Devesa *et al.*, 2018; Magnusson *et al.*, 2018; Polyzos *et al.*, 2018; Law *et al.*, 2019; Neves *et al.*, 2023; Schouten *et al.*, 2023).

However, these are all correlation studies, and the relation between oocyte number and live birth rates therefore may not be necessarily pointing to causality. Such causality may be demonstrated by randomized FSH dosing studies, with the assumption that the number of oocytes can be directly influenced by the dose of gonadotrophins. According to RCTs and related meta-analyses, there is evidence that a higher FSH starting dose results in more oocytes recovered in low, normal, and high responder groups (Lensen *et al.*, 2024).

In RCTs comparing different gonadotrophin types or different dosages of gonadotropins, differences between study groups in number of oocytes were rather limited (1–2 oocytes difference) (Andersen *et al.*, 2017; Van Tilborg *et al.*, 2017; Ishihara *et al.*, 2021; Qiao *et al.*, 2021). In these studies, this higher oocyte number did not translate into higher live birth rates per fresh transfer, whereas non-significant but possibly clinically relevant trends toward higher cumulative live birth rates were reported with higher gonadotropin starting doses in expected low responders in some, but not in other, RCTs (Van Tilborg *et al.*, 2017; Liu *et al.*, 2022). Whether larger differences in oocyte numbers within the context of randomized comparisons do create differences in cumulative live birth prospects, it is suggested in the OPTIMIST study where higher oocyte numbers doubled the proportion of couples having a second or third child (Schouten *et al.*, 2022).

### Clinical implications

Up till recently, clinicians had to select a dose based on their own subjective experience and interpretation of predictive parameters like age, ORT, BMI, etcetera. Over the last years, different algorithms have been developed that can be used to calculate the gonadotropin starting dose with the intent to optimize reproductive outcome and to reduce the risk of OHSS (Popovic‐Todorovic *et al.*, 2003; La Marca *et al.*, 2013; Lan *et al.*, 2013; La Marca and Sunkara, 2014; Olivennes *et al.*, 2015; Allegra *et al.*, 2017; Magnusson *et al.*, 2017). For follitropin delta, a comparable dosing strategy restricted to follitropin delta has been developed (Andersen *et al.*, 2017). However, due to the unique pharmaco-kinetic and -dynamic profile of follitropin delta, it cannot be compared to other gonadotropin preparations. Therefore, we developed new models: an optimal model, that has more stable predictions in different settings, and a clinically feasible model, which is easy to use in clinical practice. The dose-calculator provides an overview of several gonadotropin dosages with the combined treatment risk per dosage, which would aid the clinician in choosing an appropriate starting dose (see Tables 5 and 6). This dose selection model should however be evaluated in clinical practice to confirm if it aids the clinician to choose an appropriate starting dose. The clinical applicability of this model is currently being evaluated after implementation in two study centers in the Netherlands.

## Conclusion

This dose selection model, consisting of easily obtainable objective patient characteristics, may aid in choosing the optimal gonadotropin alpha/beta stimulation dose for each individual patient. This optimal dose would help to minimize treatment risks, whilst maintaining effectiveness and could potentially reduce patient burden and treatment costs.

## Supplementary Material

dmae032_Supplementary_Data

## Data Availability

The data underlying this article were provided by several research groups with permission. Data will be shared on request to the corresponding author with permission of all participating research groups.
