# Supplementary material for: Development and validation of a gonadotropin dose selection model for optimized ovarian stimulation in IVF/ICSI: an individual participant data meta-analysis
Source: Hum Reprod Update. 2024 Dec 20;31(2):116–32. doi: 10.1093/humupd/dmae032 (PMC11879166; doi:10.1093/humupd/dmae032)
Supplement: dmae032_Supplementary_Data [file dmae032_supplementary_data.docx]

Supplementary Data File S1

**The detailed systematic search strategy**

Study eligibility

*Type of studies:* All published and unpublished randomized controlled trials (RCT) are eligible for inclusion. Other study types will be excluded.

*Participants:* Subfertile women undergoing IVF/ICSI.

*Intervention and comparator:*

Studies must have determined at least one ovarian reserve test (bFSH, AMH or AFC).

Following the inclusion criteria used in Lensen et al (2018):

- Studies comparing ovarian stimulation doses with each other or comparing ORT-based gonadotropin dosing versus an alternative dosing policy (using an algorithm, individualized approach or a uniform dose), will be included.
  - For example: standard versus adjusted dose.
  - For example: standard versus individualized dosing
  - For example: individualized versus individualized dosing.
- Studies comparing doses of human menopausal gonadotropin (HMG), which contains both FSH and luteinizing hormone, will also be included only if both study arms use the same preparation in the same ratio.
  - For example: 150 FSH vs 225 FSH
  - For example: 150 FSH/150 hMG vs 225 FSH/225 hMG
  - For example: 100 hMG vs 150 hMG
- Studies that do not explicitly fall into the above described types of design, but of which it is possible to interpret and analyze data in such a way that they are equivalent, will also be included.
- Studies using follitropin alfa or follitropin beta

Following the exclusion criteria used in Lensen et al (2018):

- Studies comparing different preparations, brands or routes of administration will be excluded. Studies comparing same dose HMG to pure FSH will be excluded. Studies comparing step-up/step-down protocols will be excluded. Studies comparing only different stimulation regimes with no difference in gonadotropin dosing will be excluded.
  - For example: 150 FSH + antagonist vs 150 FSH + agonist
  - For example: letrozol + FSH vs FSH alone
  - For example: 150 hMG vs 150 FSH
- Studies comparing medications other than gonadotropin alfa, gonadotropin beta or hMG will be excluded.
  - For example: Corifollitropine alfa
  - For example: Follitropin delta
- Studies including only or mostly women with polycystic ovary syndrome (PCOS) were excluded.
- Studies including only or mostly women with a planned freeze all strategy were excluded.
  - For example: oocyte vitrification of social or oncology reasons
  - For example: PGT

*Outcomes:*

- Preferably live birth and/or ongoing pregnancy or at least clinical pregnancy (for definitions of outcome, see Supplementary File S3 for the definitions used.
- Preferably at least one treatment risk element (moderate/severe OHSS or preventative measures for OHSS including cycle cancellation for hyper response, freeze all, coasting and/or GnRH agonist triggering).

*Timing:*

Patients were followed-up until the result after the fresh embryo transfer of the first IVF/ICSI cycle after randomization is known. Subsequent cryopreserved embryo cycles or additional fresh IVF/ICSI cycles were not analyzed.

*Setting:*

Women being treated for their subfertility in secondary or tertiary hospitals with fertility departments or fertility clinics world-wide.

*Search:*

This study is the IPD version of the systematic review on ovarian stimulation (Lensen et al. 2018). Therefore, the search strategy is identical to the one used by Lensen. That search yielded 2422 unique articles up until 27-07-2017. Eventually, 20 articles were included.

The search was updated on 07-11-2018. Since some databases are not equipped to search from an exact date, but only a year, we choose to perform the whole search from 01-01-2017 to 07-11-2018. The electronic databases Gynaecology and Fertility Group (CGF) Specialised Register of Controlled Trials, The Cochrane Central Register of Studies Online (CRSO), MEDLINE, Embase and CINAHL will be used to identify articles. Other electronic sources as the Latin American and Caribbean of Health Sciences Information System (LILACS), the Database of Abstracts of Reviews of Effects (DARE), the institute for Scientific Information (ISI) Web of Knowledge, the World Health Organization (WHO) International Clinical Trials Registry Platform (ICTRP), clinicaltrials.gov and OpenGrey will be used as well. Relevant reference lists were screened for potential eligible studies as well. For details on the search strategies, we refer to the appendices of the original meta-analysis and Supplementary File S2 (Lensen et al. 2018).

After removal of duplicates, 637 articles were screened based on title/abstract independently by two reviewers (N.S. and R.W.) using Covidence systematic review software. 619 articles were excluded and 18 articles were assessed full-text for eligibility. Disagreements were resolved by discussion with a third and fourth reviewer (F.B. and H.T.). Six potential studies were added to this IPD-MA compared to the Cochrane systematic review. The selection process is documented using the “PRISMA IPD flow diagram” (Moher et al., 2009).

Inclusion and exclusion of studies

The authors of the 20 original studies and the 6 other studies were contacted to determine if their studies were eligible to participate in the IPD. 12/20 accepted and shared their participant data (Allegra et al. 2017; Bastu et al. 2016; Jayaprakasan et al. 2010; Klinkert et al. 2005; Lan et al. 2013; Lefebvre et al. 2015; Magnusson et al. 2017; Olivennes et al. 2015; Oudshoorn et al. 2017; Popovic‐Todorovic et al. 2003; Tasker et al. 2010; Van Tilborg et al. 2017). 2/20 studies used follitropin delta, which could not be converted to standard international units and could therefore not be compared to the other studies (Arce et al. 2014; Nyboe Andersen et al. 2017). We decided to exclude those studies. 3/20 study’s authors did not response after several attempts (Cavagna M et al. 2006; Tan et al. 2005; Yong et al. 2003). 2/20 authors responded that the original data was no longer available (Harrison et al. 2001; Out et al. 2004). And 1/20 studies reported that because the study was published before 2007, it was considered out of the scope of their data sharing policy and they were therefore not willing to share the data (Hoomans, Mulder, and Group 2002).

Of the 6 studies yielded by the updated search, 2/6 were still recruiting, but were interested to participate in a future update of this IPD (Lobato-Pascual et al. 2017; NCT02430740). After correspondence with the author, 1/6 articles did not meet the inclusion criteria and was excluded (NCT02915900 2016). 2/6 correspondence authors did not respond to several invitations to join the collaboration (NCT03402620 ; Thomas et al. 2018). 1/6 studies was initially included and data was received. However, after irregularities during the consistency checks, the author did not respond. Therefore, we were unable to use the data and excluded the study from the analysis.

We additionally included a study published after completing the systematic search (Friis Petersen et al. 2019). And we included another study found when reading the thesis manuscript of Klinkert, which is finished but unpublished (Klinkert et al. Unpublished).

# Supplementary Data File S2

**Search strategiess**

Cochrane Gynaecology and Fertility specialised register search strategy

PROCITE platform

Searched from 01-01-2017 until 05-11-2018

Results: 51

Search string #1 (in keywords and in record title apart, combined with OR):

“individualised protocol” or “individualized protocol” or “CONSORT” or ”dosing regimen“ or ”fixed dose protocol“ or ”fixed protocol“ or ”standard dose“ or ”standard protocol“ or ”standard treatment algorithm“ or ”standard treatment algorythm“ or ”standard treatment“ or ”standard stimulation“ or ”standard schedule“ or ”prediction“ or ”AMH“ or ”anti mullerian hormone“ or ”antral follicles“ or ”ovarian reserve“ or ”ovarian response“ or ”ovarian responsiveness“ or ”flexible protocol“ or ”patient-centeredness“ or ”patient orientated“ or ”dose response relationship“ or ”dose-response study“

Search string #2 (in keywords and in record title apart, combined with OR):

“FSH” or “FSH dosage” or “FSH ovarian reserve test” or “follicle stimulating hormone” or “follitropin” or “follitrophin beta” or “Follitropin A” or “follitropin alfa” or “Follitropin B” or “follitropin beta” or “rFSH” or “rhFSH” or “recombinant follicle-stimulating hormone” or “recombinant FSH” or “recombinant hFSH” or “Gonal-F” or “urofollitropin” or “pergonal” or “pergonol” or “Bravelle” or “menotropin” or “menotrophin” or “Menopur” or “highly purified FSH” or “highly purified urinary FSH” or “hp-FSH” or “hp uFSH”

Search string #3:

#1 AND #2 AND from 01-01-2017 until 05-11-2018

*51 results (no duplicates)*

| #1 | FSH | 3692 |
| --- | --- | --- |
| #2 | FSH dosage | 17 |
| #3 | “follicle stimulating hormone” | 3008 |
| #4 | “follitropin” | 1548 |
| #5 | “follitrophin beta” | 1 |
| #6 | “Follitropin A” | 5 |
| #7 | “follitropin alfa” | 73 |
| #8 | “Follitropin B” | 2 |
| #9 | “follitropin beta” | 43 |
| #10 | “rFSH” | 456 |
| #11 | “rhFSH” | 157 |
| #12 | “recombinant follicle-stimulating hormone” | 258 |
| #13 | “recombinant FSH” | 588 |
| #14 | “recombinant hFSH” | 3 |
| #15 | “Gonal-F” | 173 |
| #16 | “urofollitropin” | 104 |
| #17 | “pergonal” | 27 |
| #18 | “pergonol” | 3 |
| #19 | “Bravelle” | 29 |
| #20 | “menotropin” | 46 |
| #21 | “menotrophin” | 30 |
| #22 | “Menopur” | 53 |
| #23 | “highly purified FSH” | 33 |
| #24 | “highly purified urinary FSH” | 43 |
| #25 | “hp-FSH” | 23 |
| #26 | “hp uFSH” | 1 |
| #27 | (“FSH” or “FSH dosage” or “FSH ovarian reserve test” or “follicle stimulating hormone” or “follitropin” or “follitrophin beta” or “Follitropin A” or “follitropin alfa” or “Follitropin B” or “follitropin beta” or “rFSH” or “rhFSH” or “recombinant follicle-stimulating hormone” or “recombinant FSH” or “recombinant hFSH” or “Gonal-F” or “urofollitropin” or “pergonal” or “pergonol” or “Bravelle” or “menotropin” or “menotrophin” or “Menopur” or “highly purified FSH” or “highly purified urinary FSH” or “hp-FSH” or “hp uFSH”):kw OR (“FSH” or “FSH dosage” or “FSH ovarian reserve test” or “follicle stimulating hormone” or “follitropin” or “follitrophin beta” or “Follitropin A” or “follitropin alfa” or “Follitropin B” or “follitropin beta” or “rFSH” or “rhFSH” or “recombinant follicle-stimulating hormone” or “recombinant FSH” or “recombinant hFSH” or “Gonal-F” or “urofollitropin” or “pergonal” or “pergonol” or “Bravelle” or “menotropin” or “menotrophin” or “Menopur” or “highly purified FSH” or “highly purified urinary FSH” or “hp-FSH” or “hp uFSH”):ti (Word variations have been searched) | 3866 |
| #28 | “individualised protocol” | 11 |
| #29 | “individualized protocol” | 11 |
| #30 | “CONSORT” | 1993 |
| #31 | ”dosing regimen“ | 1883 |
| #32 | ”fixed dose protocol“ | 10 |
| #33 | ”fixed protocol“ | 51 |
| #34 | ”standard dose“ | 3249 |
| #35 | ”standard protocol “ | 1008 |
| #36 | ”standard treatment algorithm“ | 2 |
| #37 | ”standard treatment algorythm“ | 1 |
| #38 | ”standard treatment“ | 7373 |
| #39 | ”standard stimulation“ | 34 |
| #40 | ”standard schedule“ | 108 |
| #41 | ”prediction“ | 10623 |
| #42 | ”AMH“ | 448 |
| #43 | ”anti mullerian hormone“ | 335 |
| #44 | ”antral follicles“ | 74 |
| #45 | ”ovarian reserve“ | 455 |
| #46 | ”ovarian response“ | 596 |
| #47 | ”ovarian responsiveness“ | 33 |
| #48 | ”flexible protocol“ | 29 |
| #49 | ”patient-centeredness“ | 97 |
| #50 | ”patient orientated“ | 75 |
| #51 | ”dose response relationship“ | 31157 |
| #52 | ”dose-response study“ | 1192 |
| #53 | (“individualised protocol” or “individualized protocol” or “CONSORT” or ”dosing regimen“ or ”fixed dose protocol“ or ”fixed protocol“ or ”standard dose“ or ”standard protocol“ or ”standard treatment algorithm“ or ”standard treatment algorythm“ or ”standard treatment“ or ”standard stimulation“ or ”standard schedule“ or ”prediction“ or ”AMH“ or ”anti mullerian hormone“ or ”antral follicles“ or ”ovarian reserve“ or ”ovarian response“ or ”ovarian responsiveness“ or ”flexible protocol“ or ”patient-centeredness“ or ”patient orientated“ or ”dose response relationship“ or ”dose-response study“):kw OR (“individualised protocol” or “individualized protocol” or “CONSORT” or ”dosing regimen“ or ”fixed dose protocol“ or ”fixed protocol“ or ”standard dose“ or ”standard protocol“ or ”standard treatment algorithm“ or ”standard treatment algorythm“ or ”standard treatment“ or ”standard stimulation“ or ”standard schedule“ or ”prediction“ or ”AMH“ or ”anti mullerian hormone“ or ”antral follicles“ or ”ovarian reserve“ or ”ovarian response“ or ”ovarian responsiveness“ or ”flexible protocol“ or ”patient-centeredness“ or ”patient orientated“ or ”dose response relationship“ or ”dose-response study“):ti (Word variations have been searched) | 58641 |
| #54 | #27 AND #53 | 531 |
| #55 | From 01-01-2017 until 05-11-2017 | 51 |

Cochrane CENTRAL Register of Studies Online (CRSO) search strategy

Web platform

Searched from 01-01-2017 until 05-11-2018

Results: 285 (257 after removal of duplicates)

| #1 | (ovar* adj2 stimulat*):TI,AB,KY | 1674 |
| --- | --- | --- |
| #2 | MESH DESCRIPTOR Embryo Transfer EXPLODE ALL TREES | 1021 |
| #3 | MESH DESCRIPTOR Fertilization in Vitro EXPLODE ALL TREES | 1946 |
| #4 | MESH DESCRIPTOR Sperm Injections, Intracytoplasmic EXPLODE ALL TREES | 508 |
| #5 | embryo*:TI,AB,KY | 5642 |
| #5 | embryo*:TI,AB,KY | 5642 |
| #6 | (vitro fertili?ation):TI,AB,KY | 2523 |
| #7 | ivf:TI,AB,KY | 4241 |
| #8 | icsi:TI,AB,KY | 1968 |
| #9 | (intracytoplasmic sperm injection*):TI,AB,KY | 1496 |
| #10 | blastocyst*:TI,AB,KY | 954 |
| #11 | infertil* or subfertil*:TI,AB,KY | 6646 |
| #12 | assisted reproducti*:TI,AB,KY | 1019 |
| #13 | poor responder*:TI,AB,KY | 545 |
| #14 | MESH DESCRIPTOR Follicle Stimulating Hormone EXPLODE ALL TREES | 1892 |
| #15 | (Follicle Stimulating Hormone*):TI,AB,KY | 2842 |
| #16 | FSH:TI,AB,KY | 3230 |
| #17 | Follistim*:TI,AB,KY | 16 |
| #18 | hpFSH:TI,AB,KY | 2 |
| #19 | (uFSH or rhFSH):TI,AB,KY | 101 |
| #20 | (rFSH or recFSH):TI,AB,KY | 409 |
| #21 | follitropin:TI,AB,KY | 1505 |
| #22 | (Gonal F):TI,AB,KY | 157 |
| #23 | (urofollitropin or pergonal or bravelle* or follitrin):TI,AB,KY | 127 |
| #24 | (optimal or optimi?ing or optimum):TI,AB,KY | 28253 |
| #25 | consort:TI,AB,KY | 598 |
| #26 | (algorithm or algorithim):TI,AB,KY | 6394 |
| #27 | regimen*:TI,AB,KY | 57877 |
| #28 | individual*:TI,AB,KY | 85329 |
| #29 | Tailor*:TI,AB,KY | 7586 |
| #30 | (hormone based):TI,AB,KY | 14 |
| #31 | (nomogram or normogram):TI,AB,KY | 453 |
| #32 | (dose* respons*):TI,AB,KY | 41440 |
| #33 | (start* dose*):TI,AB,KY | 1669 |
| #34 | (start* dosage*):TI,AB,KY | 54 |
| #35 | (fixed dose*):TI,AB,KY | 4925 |
| #36 | (higher dose*):TI,AB,KY | 5643 |
| #37 | (total dose* or total dosage*):TI,AB,KY | 3729 |
| #38 | (standard dosage*):TI,AB,KY | 215 |
| #39 | (standard dose* or standard dosing):TI,AB,KY | 3649 |
| #40 | (increas* adj2 dose*):TI,AB,KY | 6573 |
| #41 | (increas* adj2 dosage*):TI,AB,KY | 679 |
| #42 | (higher dosage*):TI,AB,KY | 608 |
| #43 | (fixed dosage*):TI,AB,KY | 144 |
| #44 | (predictive or prediction*):TI,AB,KY | 30517 |
| #45 | predictor*:TI,AB,KY | 24195 |
| #46 | (Anti mullerian hormone):TI,AB,KY | 311 |
| #47 | AMH:TI,AB,KY | 368 |
| #48 | (Antral follicle count*):TI,AB,KY | 179 |
| #49 | AFC:TI,AB,KY | 143 |
| #50 | (Antimullerian Hormone):TI,AB,KY | 59 |
| #51 | (ovarian reserve*):TI,AB,KY | 414 |
| #52 | (ovarian respons*):TI,AB,KY | 572 |
| #53 | calculator:TI,AB,KY | 329 |
| #54 | (hormone stratif*):TI,AB,KY | 2 |
| #55 | patient* adj2 orientated:TI,AB,KY | 45 |
| #56 | (patient centered or patient centred):TI,AB,KY | 2069 |
| #57 | #1 OR #2 OR #3 OR #4 OR #5 OR #6 OR #7 OR #8 OR #9 OR #10 OR #11 OR #12 OR #13 | 12542 |
| #58 | #14 OR #15 OR #16 OR #17 OR #18 OR #19 OR #20 OR #21 OR #22 OR #23 | 4899 |
| #59 | #24 OR #25 OR #26 OR #27 OR #28 OR #29 OR #30 OR #31 OR #32 OR #33 OR #34 OR #35 OR #36 OR #37 OR #38 OR #39 OR #40 OR #41 OR #42 OR #43 OR #44 OR #45 OR #46 OR #47 OR #48 OR #49 OR #50 OR #51 OR #52 OR #53 OR #54 OR #55 OR #56 | 259803 |
| #60 | #57 AND #58 AND #59 | 984 |
| #61 | From 01-01-2017 untill 05-11-2018 | 285 |

MEDLINE search strategy

OVID platform

Searched from 01-01-2017 until 05-11-2018

Results: 44 (no duplicates)

| #1 | exp embryo transfer/ or exp fertilization in vitro/ or exp sperm injections, intracytoplasmic/ |  |
| --- | --- | --- |
| #2 | embryo transfer$.tw. |  |
| #3 | in vitro fertili?ation.tw. |  |
| #4 | ivf-et.tw. |  |
| #5 | icsi.tw. |  |
| #5 | intracytoplasmic sperm injection$.tw. |  |
| #6 | ivf.tw. |  |
| #7 | (ovar$ adj2 stimulat$).tw. |  |
| #8 | exp ovulation induction/ or superovulation/ |  |
| #9 | ovari$ hyperstimulation.tw. |  |
| #10 | ovulation induction.tw. |  |
| #11 | superovulation.tw. |  |
| #12 | COH.tw. |  |
| #13 | 14 COS.tw. |  |
| #14 | (infertil$ or subfertil$).tw. |  |
| #15 | assisted reproducti$.tw. |  |
| #16 | assisted conception.tw. |  |
| #17 | poor responder$.tw. |  |
| #18 | normal responder$.tw. |  |
| #19 | or/1-19 |  |
| #20 | exp follicle stimulating hormone/ or exp follicle stimulating hormone, beta subunit/ or exp glycoprotein hormones, alpha subunit/ or exp menotropins/ or exp urofollitropin/ |  |
| #21 | Follicle Stimulating Hormone$.tw. |  |
| #22 | fsh.tw. |  |
| #23 | (rFSH or recFSH).tw. |  |
| #24 | (uFSH or rhFSH).tw. |  |
| #25 | (hpFSH or pFSH).tw. |  |
| #26 | follitropin.tw. |  |
| #27 | Gonal F.tw. |  |
| #28 | (menotropin$ or menopur).tw. |  |
| #29 | (urofollitropin or pergonal or bravelle* or follitrin).tw. |  |
| #30 | Follistim*.tw. |  |
| #31 | or/21-31 |  |
| #32 | 20 and 32 |  |
| #33 | (optimal or optimi?ing).tw. |  |
| #34 | 35 consort.tw. |  |
| #35 | (algorithm or algorithim).tw. |  |
| #36 | regimen$.tw. |  |
| #37 | individual$.tw. |  |
| #38 | personal*.tw. |  |
| #39 | patient specific.tw. |  |
| #40 | Tailor$.tw. |  |
| #41 | hormone based.tw. |  |
| #42 | (nomogram or normogram).tw. |  |
| #43 | (dose$ adj3 respons$).tw. |  |
| #44 | (dosing adj3 respons$).tw. |  |
| #45 | (dosage$ adj3 respons$).tw. |  |
| #46 | (start$ adj2 dose$).tw. |  |
| #47 | (start$ adj3 dosage$).tw. |  |
| #48 | fixed dose$.tw. |  |
| #49 | 50 higher dose$.tw. |  |
| #50 | (total dose or total dosage).tw. |  |
| #51 | standard dosage$.tw. |  |
| #52 | (standard dose$ or standard dosing).tw. |  |
| #53 | (increas$ adj2 dose$).tw. |  |
| #54 | (increas$ adj2 dosage$).tw. |  |
| #55 | higher dosage$.tw. |  |
| #56 | fixed dosage$.tw. |  |
| #57 | (predictive or prediction$).tw. |  |
| #58 | predictor.tw. |  |
| #59 | Anti mullerian hormone.tw. |  |
| #60 | AMH.tw. |  |
| #61 | Antral follicle count$.tw. |  |
| #62 | AFC.tw. |  |
| #63 | Antimullerian Hormone.tw. |  |
| #65 | ovarian reserve$.tw. |  |
| #66 | ovarian respons$.tw. |  |
| #67 | calculator.tw. |  |
| #68 | optimum.tw. |  |
| #69 | hormone stratif$.tw. |  |
| #70 | (patient$ adj2 orientated).tw. |  |
| #71 | (patient centered or patient centred).tw. |  |
| #72 | or/34-71 |  |
| #73 | randomized controlled trial.pt. |  |
| #74 | controlled clinical trial.pt. |  |
| #75 | randomized.ab. |  |
| #76 | randomised.ab. |  |
| #77 | placebo.tw. |  |
| #78 | clinical trials as topic.sh. |  |
| #79 | randomly.ab. |  |
| #80 | trial.ti. |  |
| #81 | (crossover or cross-over or cross over).tw. |  |
| #82 | or/73-81 |  |
| #83 | 83 exp animals/ not humans.sh. |  |
| #84 | 82 not 83 |  |
| #85 | 33 and 72 and 84 | 694 |
| #86 | From 01-01-2017 until 05-11-2018 | 44 |

Embase search strategy

OVID platform

Searched from 01-01-2017 until 07-11-2018

Results: 244 (240 after removal of duplicates)

| #1 | exp embryo transfer/ or exp fertilization in vitro/ or exp intracytoplasmic sperm injection/ |  |
| --- | --- | --- |
| #2 | embryo$ transfer$.tw. |  |
| #3 | in vitro fertili?ation.tw. |  |
| #4 | icsi.tw. |  |
| #5 | intracytoplasmic sperm injection$.tw. |  |
| #6 | (blastocyst adj2 transfer$).tw. |  |
| #7 | ivf.tw. |  |
| #8 | assisted reproduct$.tw. |  |
| #9 | ovulation induc$.tw. |  |
| #10 | superovulat$.tw. |  |
| #11 | COH.tw. |  |
| #12 | infertil$.tw. |  |
| #13 | subfertil$.tw. |  |
| #14 | (ovari$ adj2 induction).tw. |  |
| #15 | exp infertility therapy/ |  |
| #16 | exp ovulation induction/ |  |
| #17 | exp ovary hyperstimulation/ |  |
| #18 | (ovar$ adj2 hyperstimulation).tw. |  |
| #19 | (ovar$ adj2 stimulat$).tw. |  |
| #20 | assisted conception.tw. |  |
| #21 | poor responder$.tw. |  |
| #22 | normal responder$.tw. |  |
| #23 | or/1-22 |  |
| #24 | exp follitropin/ |  |
| #25 | exp urofollitropin/ |  |
| #26 | Follicle Stimulating Hormone$.tw. |  |
| #27 | fsh.tw. |  |
| #28 | (rFSH or recFSH).tw. |  |
| #29 | (uFSH or rhFSH).tw. |  |
| #30 | (hpFSH or pFSH).tw. |  |
| #31 | follitropin.tw. |  |
| #32 | Gonal F.tw. |  |
| #33 | (menotropin$ or menopur).tw. |  |
| #34 | (urofollitropin or pergonal or bravelle* or follitrin).tw. |  |
| #35 | Follistim*.tw. |  |
| #36 | or/24-35 |  |
| #37 | 23 and 36 |  |
| #38 | (optimal or optimi?ing).tw. |  |
| #39 | consort.tw. |  |
| #40 | (algorithm$ or algorithim$).tw. |  |
| #41 | regimen$.tw. |  |
| #42 | (predictive or prediction).tw. |  |
| #43 | individual$.tw. |  |
| #44 | personal*.tw. |  |
| #45 | patient specific.tw. |  |
| #46 | 46 Tailor$.tw. |  |
| #47 | hormone based.tw. |  |
| #48 | (nomogram or normogram).tw. |  |
| #49 | (dose$ adj3 respons$).tw. |  |
| #50 | (dosing adj3 respons$).tw. |  |
| #51 | (dosage$ adj3 respons$).tw. |  |
| #52 | (start$ adj2 dose$).tw. |  |
| #53 | (start$ adj3 dosage$).tw. |  |
| #54 | fixed dose$.tw. |  |
| #55 | higher dose$.tw. |  |
| #56 | (total dose or total dosage).tw. |  |
| #57 | standard dosage$.tw. |  |
| #58 | (standard dose$ or standard dosing).tw. |  |
| #59 | (increas$ adj2 dose$).tw. |  |
| #60 | (increas$ adj2 dosage$).tw. |  |
| #61 | higher dosage$.tw. |  |
| #62 | fixed dosage$.tw. |  |
| #63 | predictor.tw. |  |
| #64 | Anti mullerian hormone.tw. |  |
| #65 | AMH.tw. |  |
| #66 | Antral follicle count$.tw. |  |
| #67 | AFC.tw. |  |
| #68 | Antimullerian Hormone.tw. |  |
| #69 | ovarian reserve$.tw. |  |
| #70 | ovarian respons$.tw. |  |
| #71 | calculator.tw. |  |
| #72 | optimum.tw. |  |
| #73 | hormone stratif$.tw. |  |
| #74 | (patient$ adj2 orientated).tw. |  |
| #75 | (patient centered or patient centred).tw. |  |
| #76 | or/38-75 |  |
| #77 | 37 and 76 |  |
| #78 | Clinical Trial/ |  |
| #79 | Randomized Controlled Trial/ |  |
| #80 | exp randomization/ |  |
| #81 | Single Blind Procedure/ |  |
| #82 | Double Blind Procedure/ |  |
| #83 | Crossover Procedure/ |  |
| #84 | Placebo/ |  |
| #85 | Randomi?ed controlled trial$.tw. |  |
| #86 | Rct.tw. |  |
| #87 | random allocation.tw. |  |
| #88 | randomly.tw. |  |
| #89 | randomly allocated.tw. |  |
| #90 | allocated randomly.tw. |  |
| #91 | (allocated adj2 random).tw. |  |
| #92 | Single blind$.tw. |  |
| #93 | 93 Double blind$.tw. |  |
| #94 | ((treble or triple) adj blind$).tw. |  |
| #95 | placebo$.tw. |  |
| #96 | prospective study/ |  |
| #97 | or/78-96 |  |
| #98 | case study/ |  |
| #99 | case report.tw. |  |
| #100 | abstract report/ or letter/ |  |
| #101 | or/98-100 |  |
| #102 | 97 not 101 |  |
| #103 | (exp animal/ or animal.hw. or nonhuman/) not (human or humans).ti.) |  |
| #104 | 102 not 103 |  |
| #105 | 77 and 104 | 1985 |
| #106 | From 01-01-2017 until 07-11-2018 | 244 |

CINAHL search strategy

Ebsco platform

Searched from 01-01-2017 until 05-11-2018

Results: 16 (no duplicates)

| #1 | (MM ”Follicle-Stimulating Hormone“) | 479 |
| --- | --- | --- |
| #2 | TX Follicle Stimulating Hormone* | 2,361 |
| #3 | TX FSH | 1,315 |
| #4 | TX rFSH or TX recFSH | 44 |
| #5 | TX rhFSH or TX hpFSH | 3 |
| #6 | TX follitropin | 35 |
| #7 | TX Gonal F | 15 |
| #8 | TX menotropin* or TX menopur | 8 |
| #9 | TX urofollitropin or TX pergonal or TX bravelle* or TX follitrin | 5 |
| #10 | TX FolliStim* | 2 |
| #11 | #1 OR #2 OR #3 OR #4 OR #5 OR #6 OR #7 OR #8 OR #9 OR #10 | 2,84 |
| #12 | TX vitro fertilisation | 6,23 |
| #13 | TX vitro fertilization | 6,23 |
| #14 | (MM ”Fertilization in Vitro“) | 3,072 |
| #15 | TX IVF or TX ICSI | 4,389 |
| #16 | TX ovari* N3 stimulat* | 871 |
| #17 | TX ovar* N3 hyperstimulat* | 763 |
| #18 | TX embryo* N3 transfer* | 2,69 |
| #19 | TX assisted reproducti* | 3,333 |
| #20 | TX (infertil* or subfertil*) | 15,27 |
| #21 | TX poor responder* | 440 |
| #22 | TX normal responder* | 87 |
| #23 | #12 OR #13 OR #14 OR #15 OR #16 OR #17 OR #18 OR #19 OR #20 OR #21 OR #22 | 22,475 |
| #24 | #11 AND #23 | 1,007 |
| #25 | TX individualised or TX individualized | 19,482 |
| #26 | TX (optimal or optimi?ing) | 68,969 |
| #27 | TX optimum | 6,317 |
| #28 | TX consort | 820 |
| #29 | TX algorithm or TX algorithim | 58,567 |
| #30 | TX regimen | 40,724 |
| #31 | TX personal* | 151,75 |
| #32 | TX patient specific | 20,87 |
| #33 | TX Tailor* | 22,388 |
| #34 | TX nomogram or TX normogram | 1,467 |
| #35 | TX dos* N3 respons* | 38,283 |
| #36 | TX start* N2 dos* | 1,418 |
| #37 | TX predictive or TX prediction or TX predictor | 207,308 |
| #38 | TX calculator | 1,309 |
| #39 | TX patient* N2 orientated | 102 |
| #40 | TX patient centered or TX patient centred | 32,218 |
| #41 | #25 OR #26 OR #27 OR #28 OR #29 OR #30 OR #31 OR #32 OR #33 OR #34 OR #35 OR #36 OR #37 OR #38 OR #39 OR #40 | 602,121 |
| #42 | (MH "Clinical Trials+") | 251,844 |
| #43 | PT Clinical trial | 86,267 |
| #44 | TX clinic* n1 trial* | 235,145 |
| #45 | TX ( (singl* n1 blind*) or (singl* n1 mask*) ) or TX ( (doubl* n1 blind*) or (doubl* n1 mask*) ) or TX ( (tripl* n1 blind*) or (tripl* n1 mask*) ) or TX ( (trebl* n1 blind*) or (trebl* n1 mask*) ) | 958,56 |
| #46 | TX randomi* control* trial* | 160,755 |
| #47 | (MH ”Random Assignment“) | 51,978 |
| #48 | TX random* allocat* | 9,493 |
| #49 | TX placebo* | 53,968 |
| #50 | (MH ”Placebos“) | 10,962 |
| #51 | (MH ”Quantitative studies“) | 21,249 |
| #52 | TX allocat* random* | 9,493 |
| #53 | #42 OR #43 OR #44 OR #45 OR #46 OR #47 OR #48 OR #49 OR #50 OR #51 OR #52 | 1,254,337 |
| #54 | #24 AND #41 AND #53 | 73 |
| #55 | From 01-01-2017 until 05-11-2018 | 16 |

LILACS search strategy

Web platform (LILACS and IBECS)

Searched from 01-01-2017 until 05-11-2018

Results: 2 (no duplicates)

Search string #1:

(tw:(ivf)) OR (tw:(fsh)) OR (tw:(rfsh)) OR (tw:(recfsh)) OR (tw:(hmg)) OR (tw:(gonadotrophin)) OR (tw:(gonadotropin))

*Results: 2890*

Search string #2:

(tw:(optimal)) OR (tw:(optimise)) OR (tw:(tailor)) OR (tw:(individual)) OR (tw:(dose)) OR (tw:(low-dose)) OR (tw:(fixed-dose)) OR (tw:(high-dose)) OR (tw:(dosage)) OR (tw:(doses)) OR (tw:(regimen)) OR (tw:(dose-response))

*Results: 80.154*

Search string #3:

(tw:(randomly)) OR (tw:(randomise)) OR (tw:(randomize)) OR (tw:(rct))

*Results: 10.394*

Search string #4 (#1 AND #2 AND #3):

(tw:((tw:(ivf)) OR (tw:(fsh)) OR (tw:(rfsh)) OR (tw:(recfsh)) OR (tw:(hmg)) OR (tw:(gonadotrophin)) OR (tw:(gonadotropin)))) AND (tw:((tw:(optimal)) OR (tw:(optimise)) OR (tw:(tailor)) OR (tw:(individual)) OR (tw:(dose)) OR (tw:(low-dose)) OR (tw:(fixed-dose)) OR (tw:(high-dose)) OR (tw:(dosage)) OR (tw:(doses)) OR (tw:(regimen)) OR (tw:(dose-response)) )) AND (tw:((tw:(randomly)) OR (tw:(randomise)) OR (tw:(randomize)) OR (tw:(rct)))) AND (instance:"regional") AND (db:("LILACS" OR "IBECS"))

*Results: 32*

Search string #5:

#4 AND from 01-01-2017 until 05-11-2018

*Results: 2*

DARE search strategy

Web platform

Searched from 01-01-2017 until 05-11-2018

Results: 0 (no duplicates)

| #1 | (ivf) | 243 |
| --- | --- | --- |
| #2 | (stimulation) | 1303 |
| #3 | (fsh) | 75 |
| #4 | (Rfsh) | 22 |
| #5 | (recFSH) | 0 |
| #6 | (HMG) | 97 |
| #7 | (gonadotrophin) | 129 |
| #8 | #1 OR #2 OR #3 OR #4 OR #5 OR #6 OR #7 | 1636 |
| #9 | (optimal) | 2294 |
| #10 | (optimise) | 142 |
| #11 | (tailor) | 66 |
| #12 | (individual) | 8772 |
| #13 | (dose) | 6775 |
| #14 | (low-dose) | 853 |
| #15 | (fixed-dose) | 177 |
| #16 | (high-dose) | 806 |
| #17 | (dosage) | 8055 |
| #18 | (doses) | 3564 |
| #19 | (regimen) | 2016 |
| #20 | (dose-response) | 1096 |
| #21 | #9 OR #10 OR #11 OR #12 OR #13 OR #14 OR #15 OR #16 OR #17 OR #18 OR #19 OR #20 | 21402 |
| #22 | (random) | 8486 |
| #23 | (randomly) | 1152 |
| #24 | (randomise) | 85 |
| #25 | (randomize) | 1 |
| #26 | (RCT) | 4484 |
| #27 | #22 OR #23 OR #24 OR #25 OR #26 | 12407 |
| #28 | #8 AND #21 AND #27 | 200 |
| #29 | From 01-01-2017 until 05-11-2018 | 0 |

ISIWeb of Knowledge search strategy

Web platform

Searched from 01-01-2017 until 05-11-2018

Results: 22 (21 after removal of duplicates)

Search string #1:

TI=((Ivf OR Stimulation OR FSH OR Rfsh OR recFSH OR HMG OR Gonadotrophin) AND (Optimal OR Optimise OR Tailor OR Individual OR Dose OR Low-dose OR Fixed-dose OR High-dose OR Dosage OR Doses OR regimen OR Dose-response) AND (random OR randomly OR randomise OR randomize OR RCT))

*Results: 161*

Search string #2

#1 AND from 01-01-2017 until 05-11-2018

*Results: 22*

WHO International Clinical Trials Registry Platform search strategy

Web platform

Searched from 01-01-2017 until 05-11-2018

Results: 86 (84 after removal of duplicates)

Search string #1

(Ivf OR Stimulation OR FSH OR Rfsh OR recFSH OR HMG OR Gonadotrophin) AND (Optimal OR Optimise OR Tailor OR Individual OR Dose OR Low-dose OR Fixed-dose OR High-dose OR Dosage OR Doses OR regimen OR Dose-response)

*Results 392*

Search string #2:

#1 AND from 01-01-2017 until 05-11-2018

*Results: 86*

ClinicalTrials.gov search strategy

Web platform

Searched from 01-01-2017 until 05-11-2018

Results: 105 (no duplicates)

Included: recruiting, not yet recruiting, active, not recruiting, completed, enrolling by invitation studies.

Excluded: withdrawn, suspended, terminated, unknown status.

Search string #1:

(ivf OR fsh OR Gonadotrophin) AND (Dose OR Low-dose OR Fixed-dose OR High-dose OR Dosage OR Doses OR regimen OR Dose-response) and (random OR randomly OR randomise OR randomize OR RCT)

*Results: 454*

Search string #2:

#1 AND from 01-01-2017 until 05-11-2018

*Results: 105*

OpenGrey search strategy

Web platform

Searched from 01-01-2017 until 05-11-2018

Results: 0 (no duplicates)

Search string #1:

(High-dose OR Dosage OR Doses OR regimen OR Dose-response) AND (random OR randomly OR randomise OR randomize OR RCT)

*Results: 44*

Search string #2:

#1 AND from 01-01-2017 until 05-11-2018

*Results: 0*

Supplementary Data File S3

**Outcome definitions and standardize methods**

**Age** in years

**Anti-Müllerian hormone** (AMH) in pmol/L

**Antral follicle count** (AFC) in number of follicles from 2-10 mm

**Basal Follicle-Stimulating Hormone** (bFSH) in international units/L

**Body mass index** (BMI) in kg/m^2^

**Cancel hyper response** as yes/no based on criteria defined by original study author

**Cancel low response** as yes/no based on criteria defined by original study author

**Cause of subfertility** as unknown, female, male, mixed or other.

**Clinical pregnancy** is defined as evidence of an intrauterine gestational sac on ultrasound or other definitive signs of pregnancy, including foetal heartbeat. Ectopic pregnancies do not count as clinical pregnancy.

**Coasting** as yes/no based on criteria defined by original study author

**Combined treatment risk** is defined as the development of moderate or severe ovarian hyperstimulation syndrome (OHSS) and/or any measure taken to prevent the development of OHSS, including cycle cancellation due to an excessive response, coasting, GnRH agonist triggering, freeze all and/or no embryo transfer to prevent the development of OHSS.

**Cryopreserved embryos** as number

**Cycle length** in days

**Development of moderate or severe OHSS** as yes/no based on criteria defined by original study author

**Dose adjustments** as yes/no

**Follicle puncture** as yes/no

**Freeze-all due to risk OHSS** as yes/no based on criteria defined by original study author

**Fresh transfer** as yes/no

**GnRH analogue** as agonist/antagonist

**Gonadotropin starting dose** (GSD) in international units
**Duration of subfertility** in months

**Gravidity** as the number of times that a patient has been pregnant

**Insemination method** as IVF/ICSI

**Live birth** is defined as the birth of at least one live baby regardless of gestational age.

**Moderate OHSS** is defined as the definition used by the original authors of the study.

**Multiple pregnancy** is defined as ongoing pregnancy with at least two foetuses.

**Number of fresh embryo’s transferred** as number

**Number of fresh embryos** as number

**Number of oocytes** as number

**Ongoing pregnancy** is defined as evidence of a gestational sec with foetal heart beat at or after ten to twelve weeks’ gestation, confirmed with ultrasound.

**Parity** as the number of times that a patient has given birth to a fetus with a gestational age of 24 weeks or more

**Previous ART cycles** as number of cycles

**Severe OHSS** is defined as the definition used by the original authors of the study.

**Total gonadotropin dose** in international units

**Total number of days of gonadotropin stimulation** in days

**Total number of embryos** as number

**Type of subfertility** as primary infertility/secondary infertility

Supplementary Data File S4

**Overview of risk of bias score of two additional studies compared to meta-analysis Lensen 2018.**


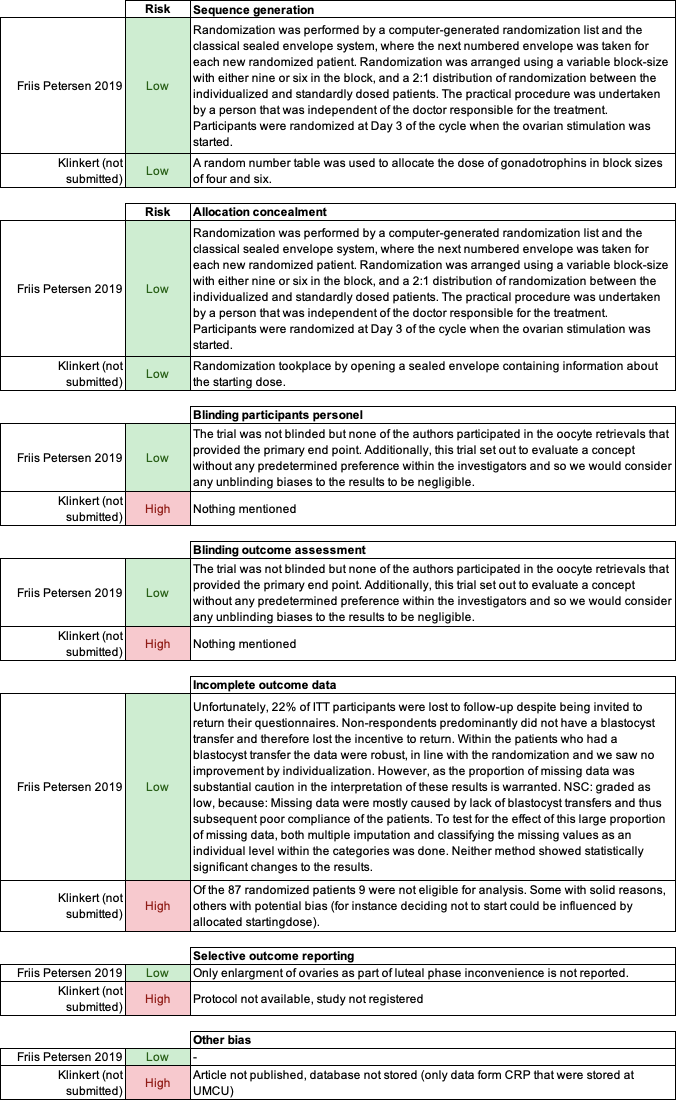


Bias was scored as low, high or unknown using the Cochrane ’Risk of bias’ assessment tool, which considers checks for bias arising from: selection (random sequence generation and allocation concealment), performance (blinding of participants and personnel), detection (blinding of outcome assessors), attrition (incomplete outcome data), reporting (selective reporting), and other causes (Higgins, Altman, and Sterne 2011).

Supplementary Data File S5

**Multiple imputation**

We used multiple imputation by chained equations for systematically and sporadically missing multilevel data, with the “mice”, “miceadds”, and “micemd” packages in R. We accounted for heterogeneity between studies in the imputation process by employing multi-level multiple imputation with the variable “Study” as cluster variable. In this approach, missing variables are imputed within each study first. If this is not feasible, it was assessed which study most closely resembled the study with missing data and imputed the missing data based on that reference. This method is a technique that helps you handle variation between studies, as we tell the analysis to consider that the data from each study might be different from one another. This method helps us impute missing data in a way that respects the differences between the studies. This allows our analysis to deal with specific study characteristics, reducing the impact of heterogeneity, resulting in more robust and accurate results. Predictive mean matching was used due to the skewness of the data distribution, a 2-level, 2-stage approach was used when there were enough events, otherwise we used a 2-level, 1-stage approach. All candidate predictors were included in the imputation model. We used log-transformations and centered around the mean to scale variables and used post-imputation transformations to prevent impossible imputations. Model convergence and imputation output were assessed by visual examination. Convergence plots are visualized below in figure 1.


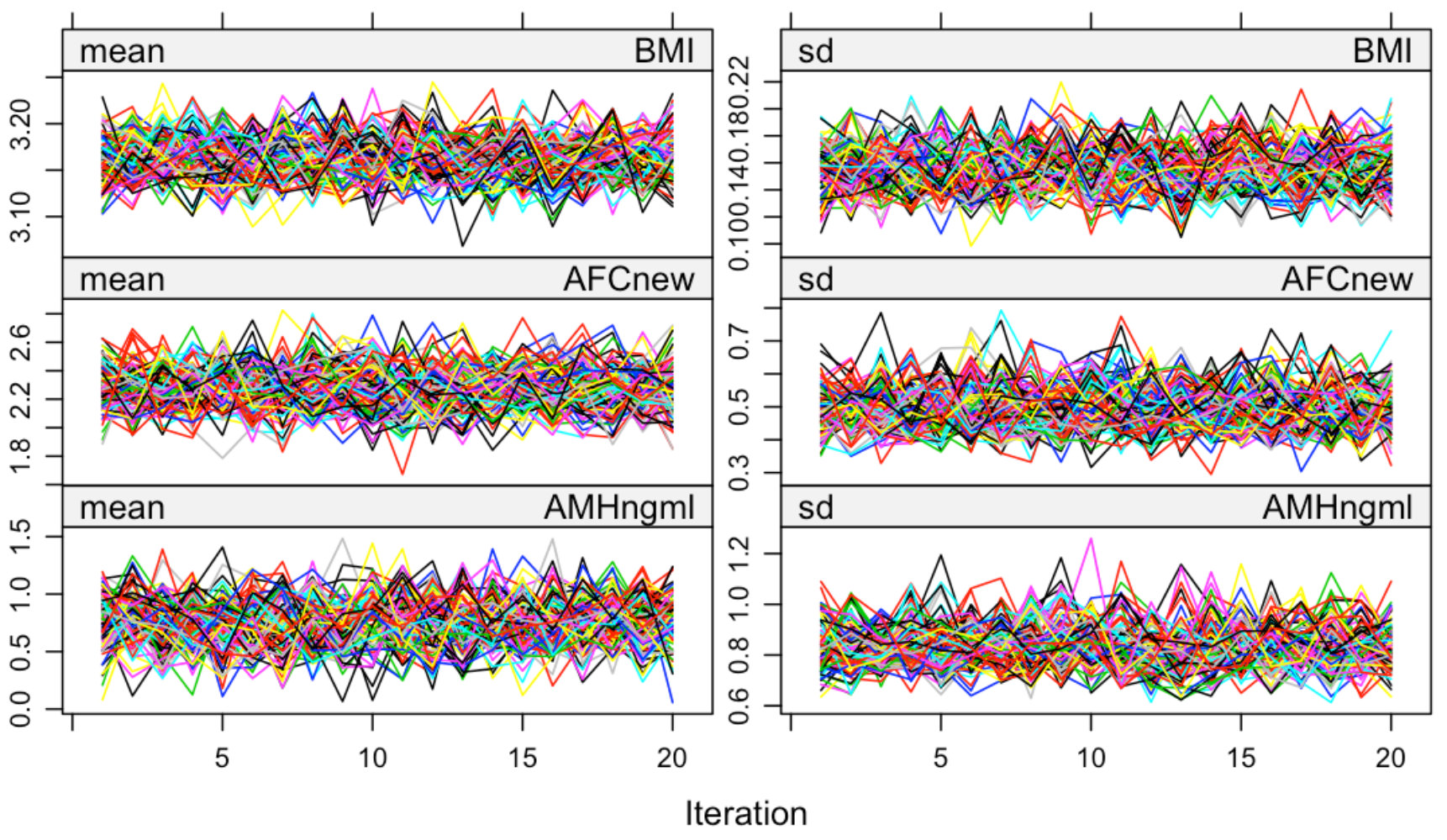


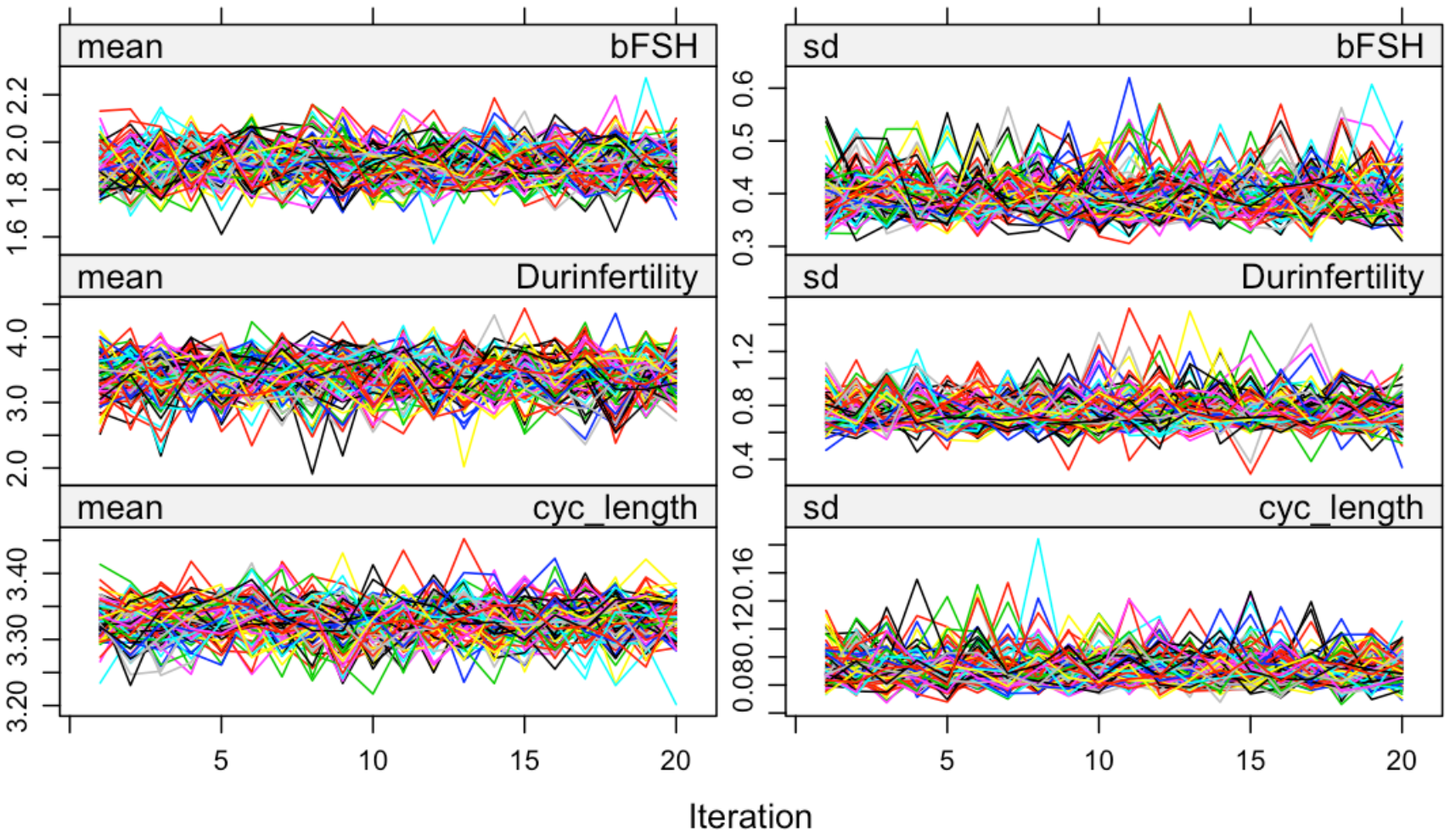


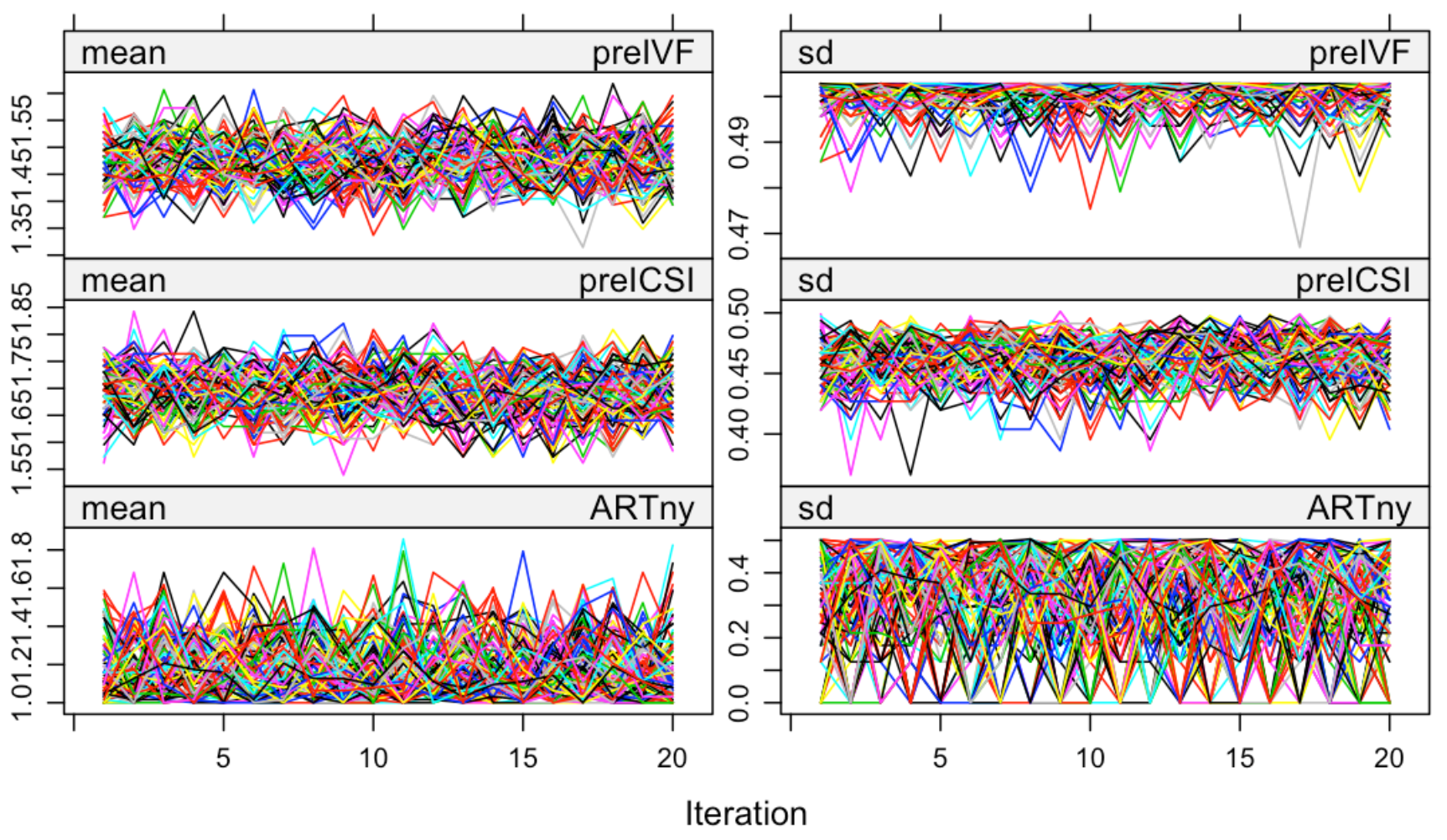


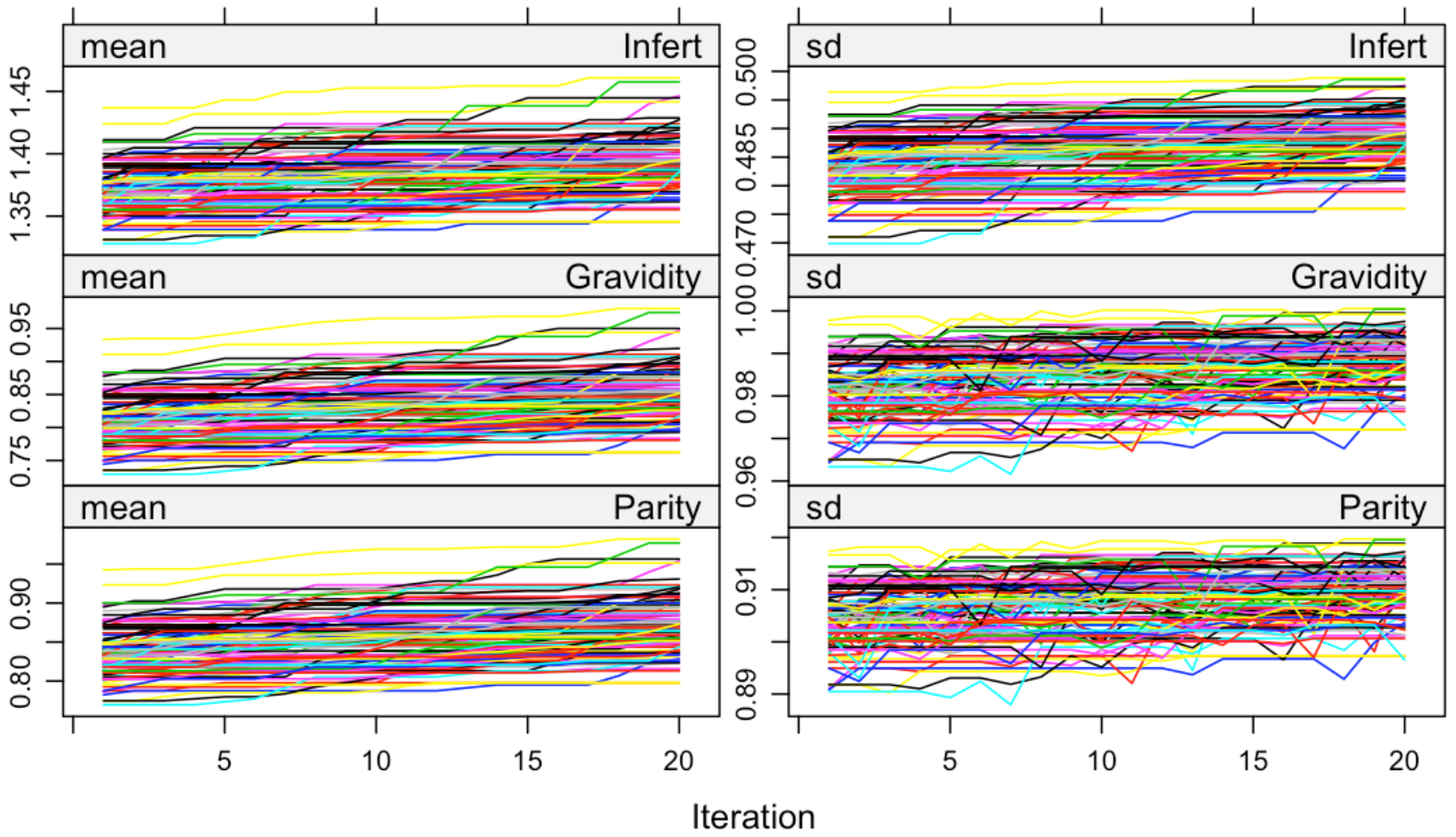


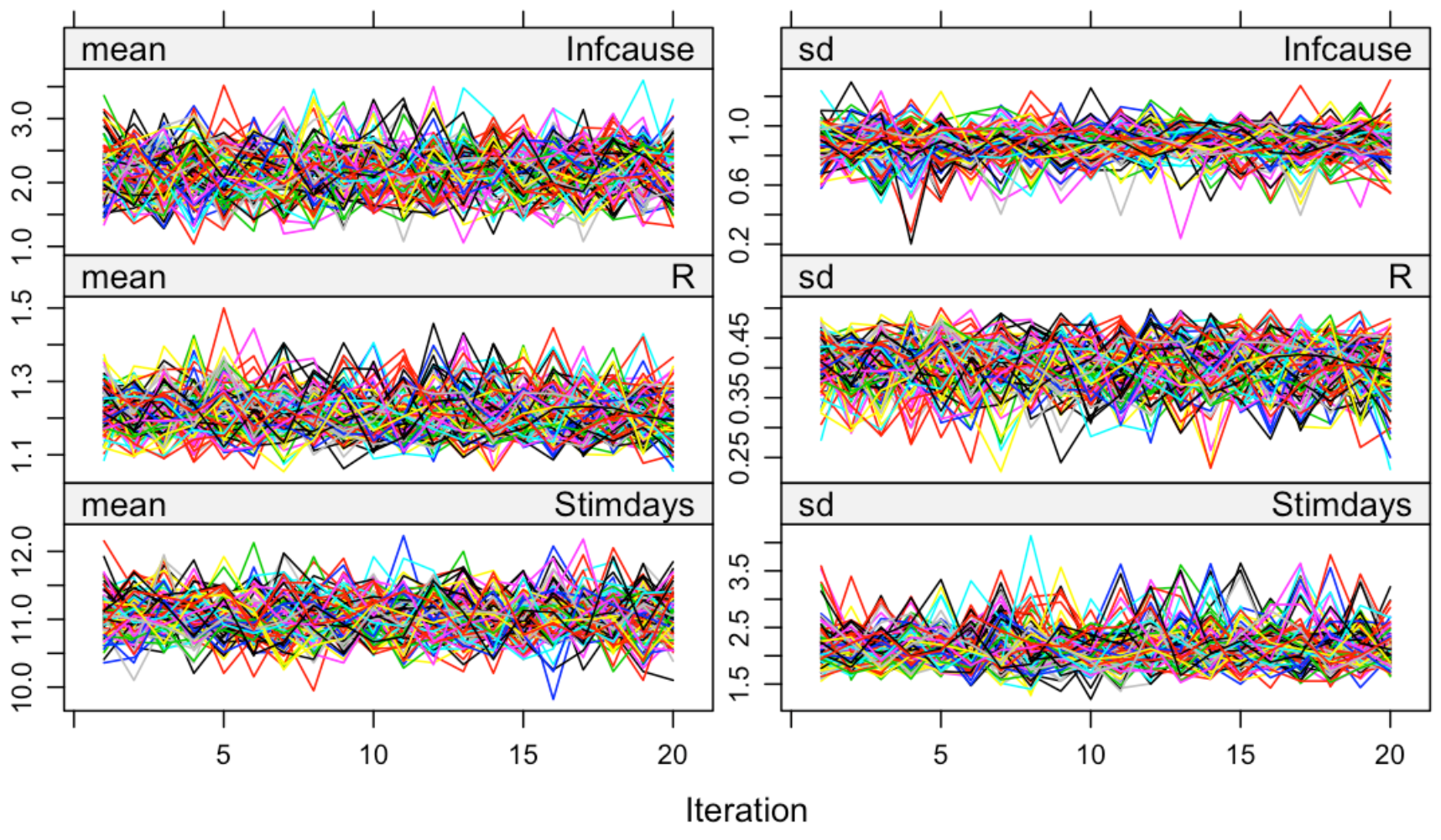


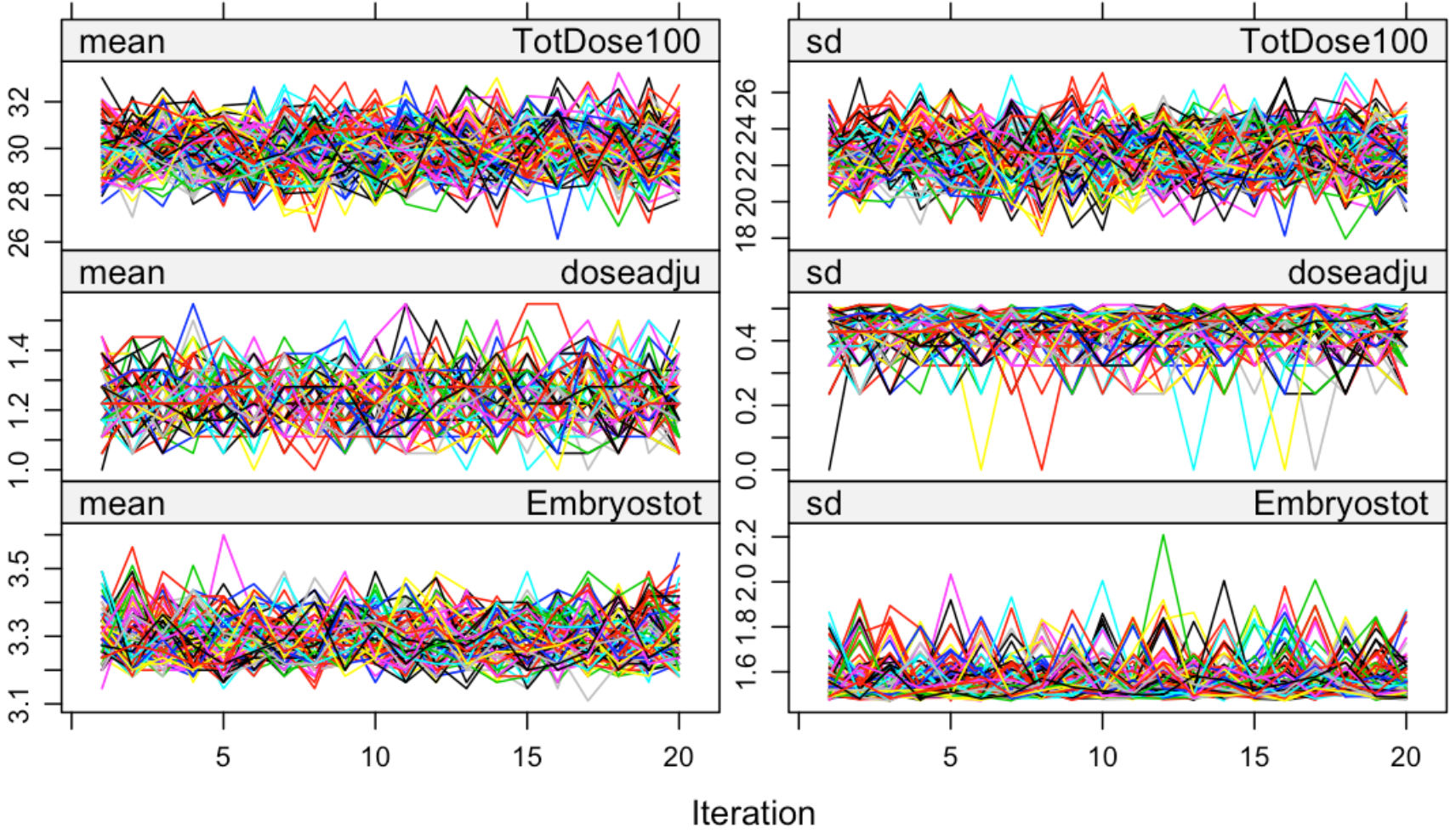


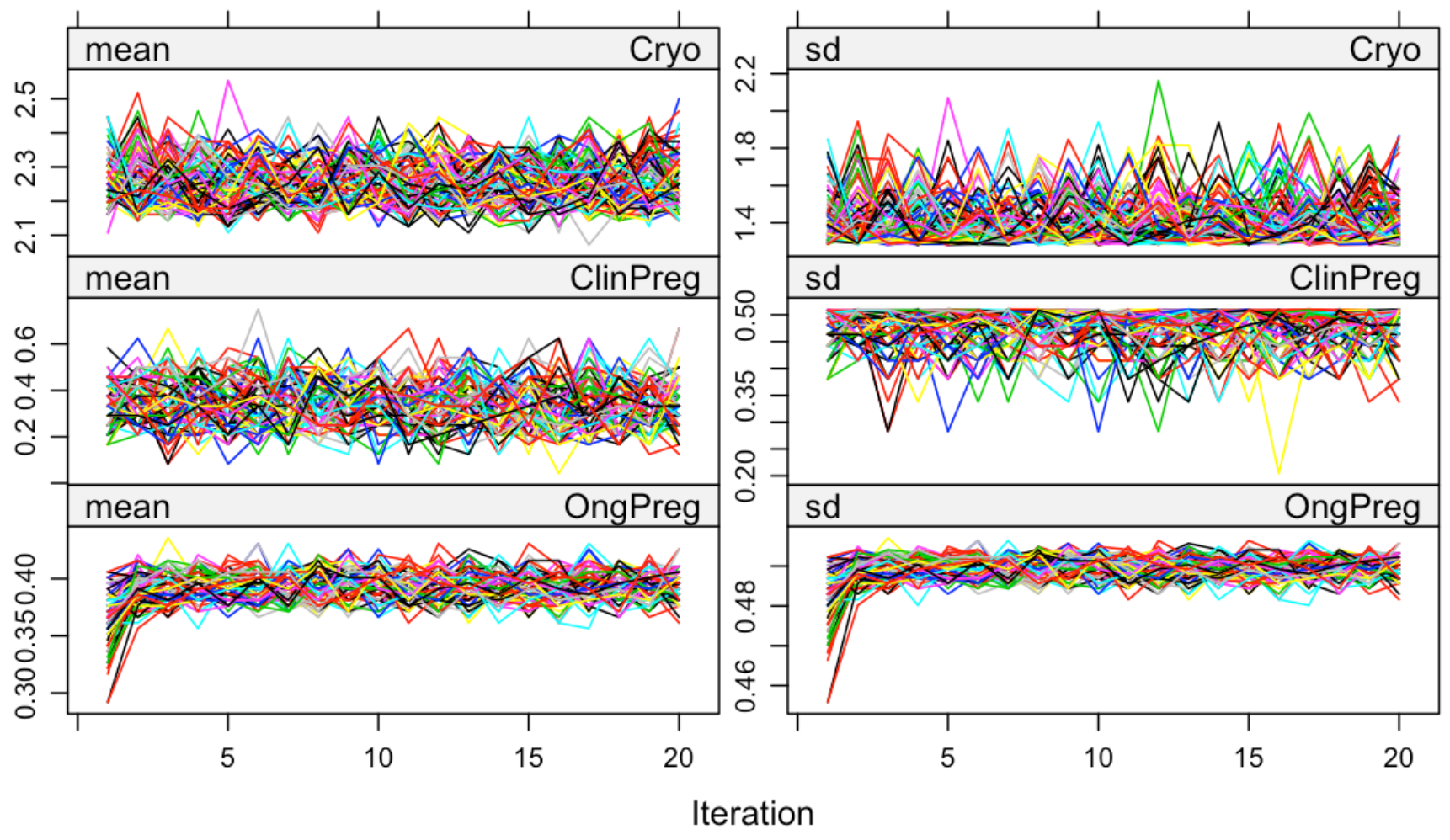


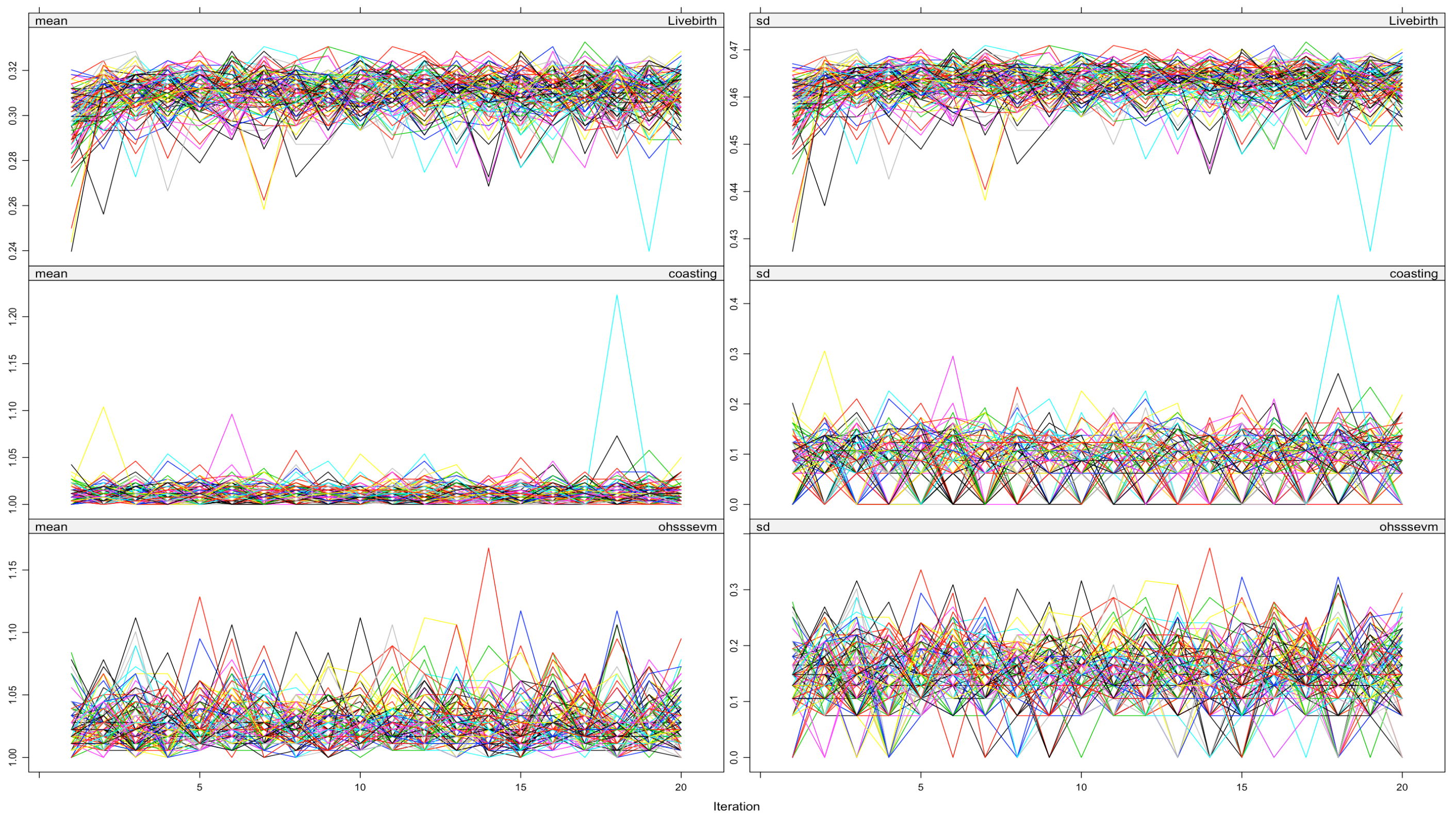


Visualisation of convergence per imputed variable using 100 imputations and 20 iterations. The mean and standard deviation of the synthetic values are plotted against the iteration number for the imputed variables. The colored streams should be intermingled with one another, without showing any definite trend. Convergences is acceptable for all variables except for gravidity, parity and type of infertility, this is due to the post-imputation changes that were necessary to avoid impossible values (for instance G1P5, of G2 with primary infertility).

*Summary plots before and after imputation*


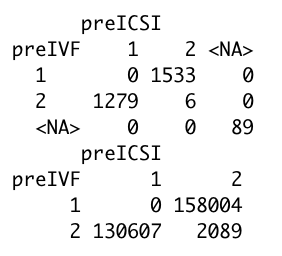

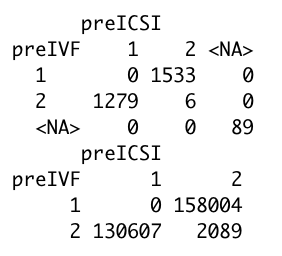


Pre- (left) and post-imputation (right) summary plots for ICSI and IVF. NA = missing.


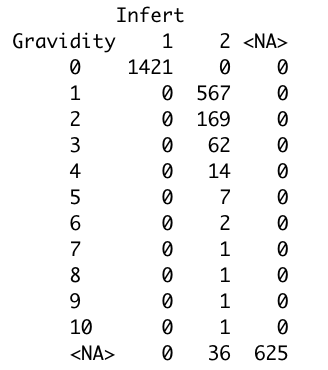

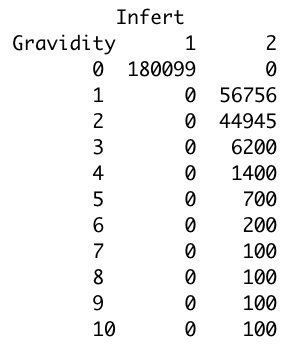


Pre- (left) and post-imputation (right) summary plots for gravidity and type of infertility. NA = missing.


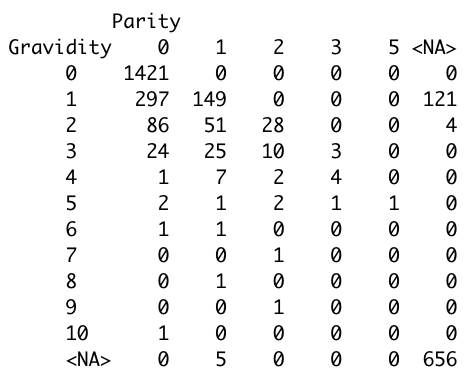

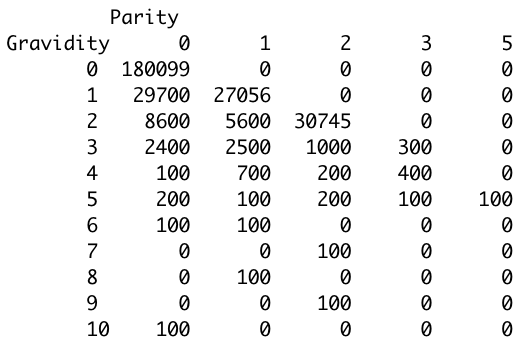


Pre- (left) and post-imputation (right) summary plots for gravidity and parity. NA = missing.


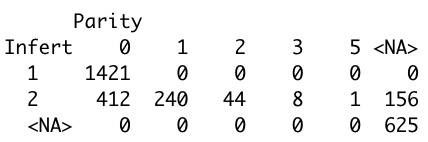

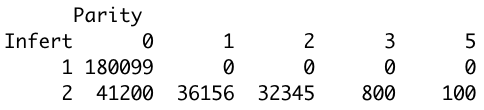


Pre- (left) and post-imputation (right) summary plots for parity and type of infertility. NA = missing.


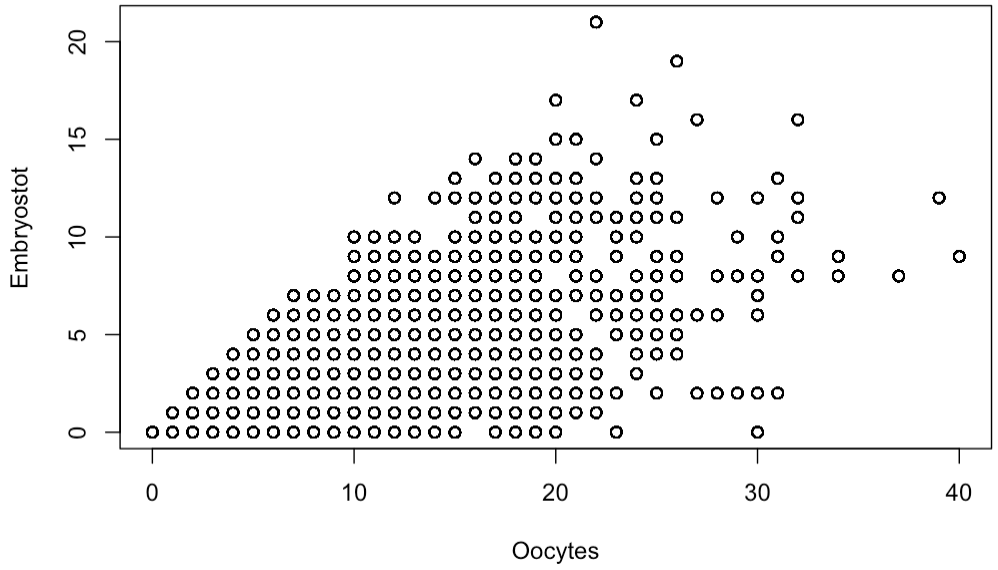


Post-imputation summary plots for number of total embryos and number of oocytes.


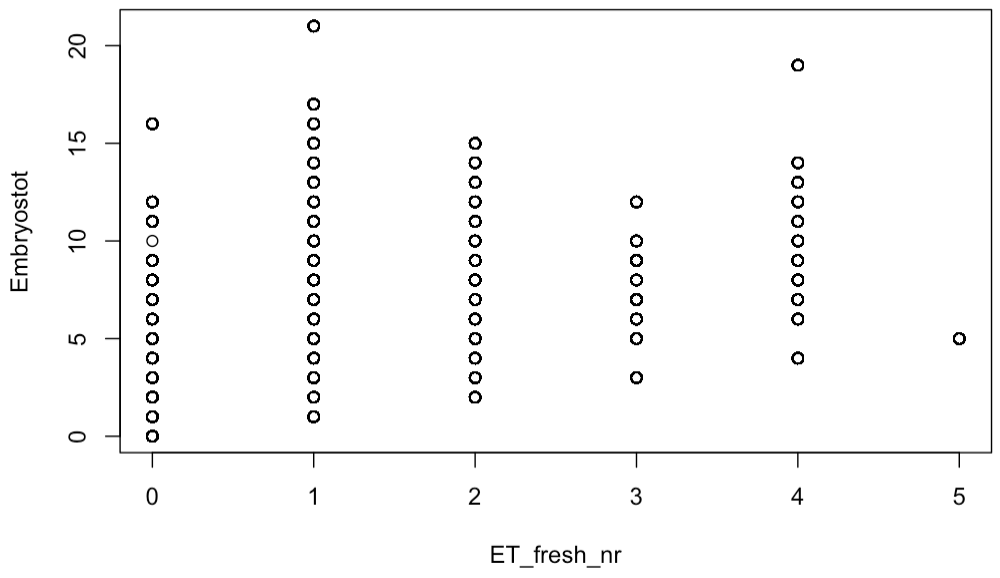


Post-imputation summary plots for number of total embryos and number of fresh embryos transferred.


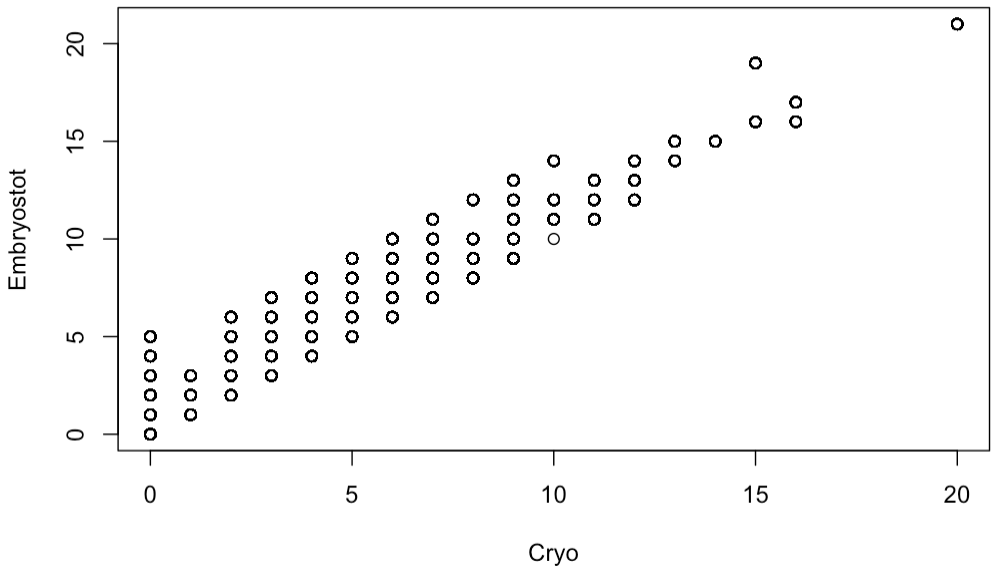


Post-imputation summary plots for number of total embryos and number of cryopreserved embryos.


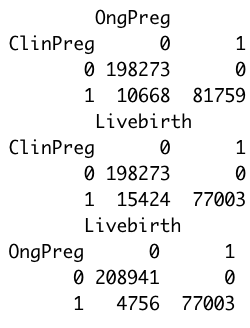


Post-imputation summary for ongoing pregnancy and clinical pregnancy, live birth and clinical pregnancy, live birth and ongoing pregnancy.

**Supplementary Data File S6**

**Visualization of the internal external cross validation process.**

Yellow depicts the studies that were used to fit a model, green depicts the study that was used to validate the model.

*Internal external cross validation*


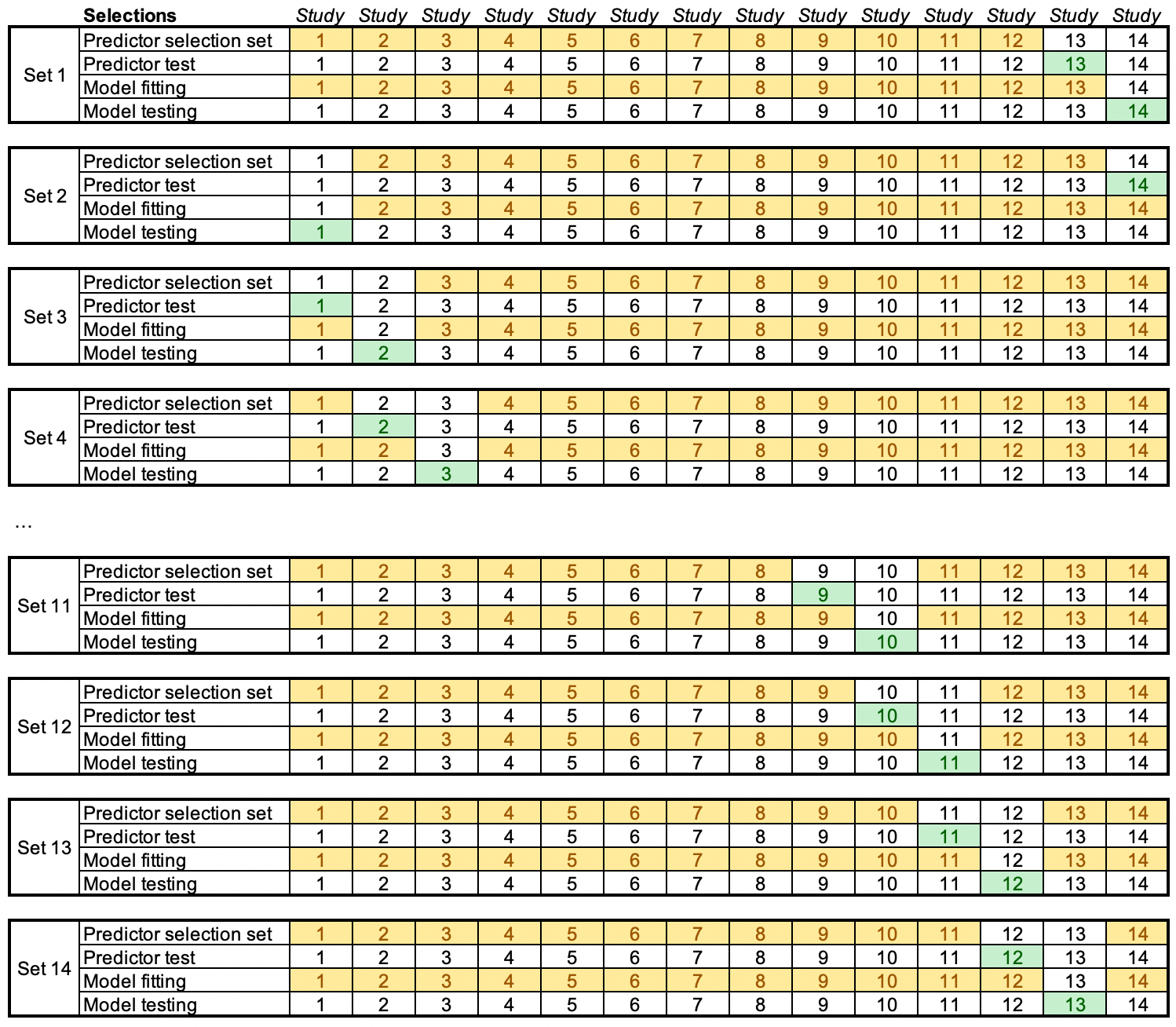


Study 1 = Allegra (2017)

Study 2 = Friis Petersen (2019)

Study 3 = Jayaprakasan (2010)

Study 4 = Klinkert (2005)

Study 5 = Lan (2013)

Study 6 = Lefebvre (2015)

Study 7 = Magnusson (2017)

Study 8 = Olivennes (2015)

Study 9 = Oudshoorn (2017)

Study 10 = Popovic-Todorovic (2003)

Study 11 = Van Tilborg (2017)

Study 12 = Bastu (2016)

Study 13 = Klinkert (unpublished)

Study 14 = Tasker (2010)

The goal of the internal-external cross validation (IECV) procedure is to assess if the model predictions hold true in different settings: for instance in different centers/populations, different periods in time, et cetera. This technique aims to reduce overfitting and improves the model’s ability to generalize. Per set we excluded 2 of the total 14 data sets. On the remaining 12 we performed backwards selection. This resulted in several exclusion steps: at each step, the least predictive variable was excluded. We tested model performance per exclusion step in the first of the two excluded studies. For example: in set 1 we performed backwards selection on all data of studies 1-12 and we tested the model per exclusion step in study 13. This was repeated until each dataset has been used once for testing model performance, resulting in 14 IECV sets. We then selected the models with the highest performance overall and used those models for final model development. In order to do so, we first fitted the model in the 12 studies + the first excluded study, and then tested the performance in the second exclusion study. We repeated this process again for all IECV sets and for all selected models. For example: in set 1 we fitted a model with the most predictive variables in studies 1-13 and validated the model in study 14.

Some studies did not have live birth or safety events: for instance study 12 and 13 did not have a safety event. These studies were used for the model fitting/development phase, but could not be used to validate the model. So there was no model validation in set 13 and set 14.

Backwards selection was performed on all candidate predictors per imputation set with generalized linear (mixed) models using the “psfmi” package in R. We utilized "Pooling and backward selection for 2 level (generalized) linear mixed models in multiply imputed datasets". In the psfmi package, we used the “random.eff = (1 | Study) option, which specifies the random effects structure for linear mixed-effects models.

We assessed model discrimination (using AUCs) and plotted calibration curves per imputation set, of which the results were pooled using Rubin’s Rules. This approximates the performance of the final model in different settings, as the individual studies represent different geographical locations, different time periods and different populations. Forest plots of the performance measures were made and summary statistics were calculated using a random effects meta-analysis estimate. This resulted in a summary AUC, summary calibration slope and summary calibration-in-the-large (CIL) per model. The final models (the optimal and clinically feasible models for both live birth and safety) were developed using a one-stage approach with all available data pooled, using accounting for clustering within studies.

The model with the highest pooled performance was chosen as the “optimal model”. Gonadotropin starting dose (GSD) was forced into the model, as it is necessary for dose selection. Age, BMI and GSD were transformed using restricted cubic splines with 3 knots to model the non-linear trend of these variables. For the interaction between age and GSD, we corrected for within and between study heterogeneity by centering around the individual study means, using current statistical practice (Riley et al. 2020). This approach helps account for differences in covariate distributions between studies, ensuring that the effects of covariates are estimated within the context of each study's unique characteristics. We only validated models in studies with at least 1 event.

For the final selected inner IECV models, we assessed on the outer IECV model discrimination (AUCs) and plotted calibration curves per imputation set, of which the results were pooled using Rubin’s Rules. We depicted the resulting performance measures per IECV set as forest plots and calculated the summary statistic of all outer IECV sets using a random effects meta-analysis estimate. This resulted in a summary AUC, summary calibration slope and summary calibration-in-the-large (CIL) per model. For the summary AUC calculation, we transformed the AUC into corresponding OR before meta-analyzing with random effects (Walter & Sinuff, 2007). The results of the internally-externally cross validated models show whether the model predictions hold true in different settings. The final models (the optimal and clinically feasible models for both live birth and safety) were developed using a one-stage approach with all available data pooled, using accounting for clustering within studies. This is important to correct for differences within and between studies.

A meta-analysis was performed using the metagen function (Rstudio). This function uses the inverse variance method for pooling estimates, which entails weighting each estimate by the inverse of its variance (1/standard error squared). This weighting considers both study size and estimate precision. Additionally, the between-study variance was estimated (tau-squared, τ²) in a random-effects model using the DerSimonian and Laird method.

We also introduced variables to the model to represent the mean age per study and mean dosage per study. In the model presented in this paper as example, we used the mean age and mean starting dose of the entire dataset. However in the model this can be adjusted. For instance, if a new center wishes to use the model but has an average population age of 37, while the dataset's mean age is 34, this can be accommodated in the model. The same can be done for mean starting dose. This way, the model can serve as a general applicable model or be tailored to better represent the characteristics of a specific target population.

Supplementary Data File S7

**Overview of integrity check.**

When informed consent is obtained, the study is registered prospectively and there was study approval from an ethical committee, integrity was deemed normal. Green means no concern regarding integrity or ethical questions. Yellow means unknown but probably not problematic. Red means concerns regarding integrity and/or ethical check.


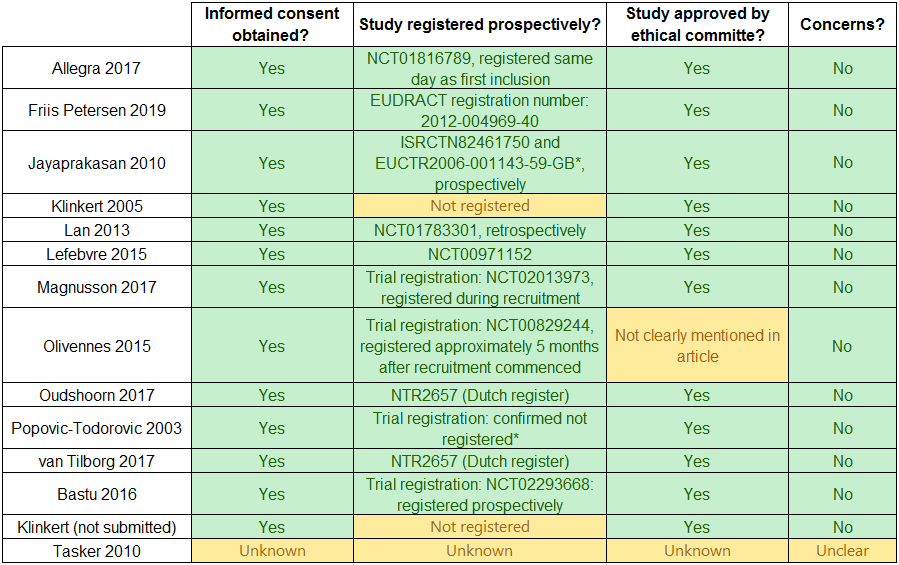


Supplementary Data File S8.

**Calibration in the large and slope of calibration curves live birth models**


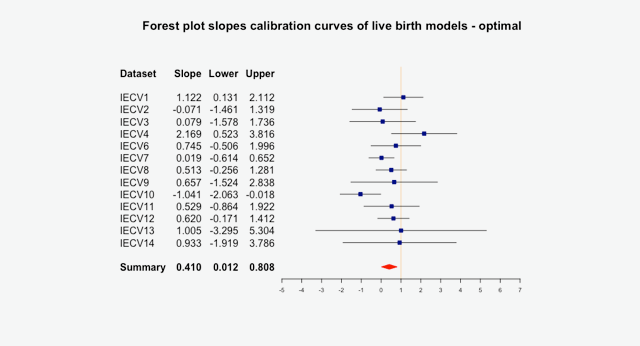


Forest plot of slopes of the optimal live birth models including age, starting dose, body mass index, antral follicle count, fertilization with IVF or ICSI and anti-Müllerian hormone. IECV = internal external cross-validation set, Lower = lower 95% of confidence interval, Upper = upper 95% of confidence interval. Summary statistic results in an overall slope of 0.410 (0.012-0.808). IECV1 results were tested in Tasker (2010), IECV2 results were tested in Allegra (2017), IECV3 results were tested in Friis Petersen (2019), IECV4 results were tested in Jayaprakasan (2010) , IECV5 results were tested in Klinkert (2005), IECV6 results were tested in Lan (2013), IECV7 results were tested in Lefebvre (2015), IECV8 results were tested in Magnusson (2017), IECV9 results were tested in Olivennes (2015), IECV10 results were tested in Oudshoorn (2017), IECV11 results were tested in Popovic-Todorovic (2003), IECV12 results were tested in Van Tilborg (2017), IECV13 results were tested in Bastu (2016), IECV14 results were tested in Klinkert (unpublished).


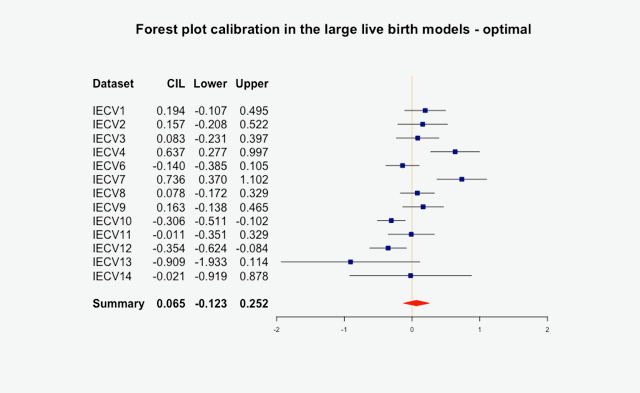


Forest plot of calibration in the large of the optimal live birth models including age, starting dose, body mass index, antral follicle count, fertilization with IVF or ICSI and anti-Müllerian hormone. IECV = internal external cross-validation set, Lower = lower 95% of confidence interval, CIL = calibration in the large Upper = upper 95% of confidence interval. Summary statistic results in an overall calibration in the large of 0.065 (-0.123 – 0.252). IECV1 results were tested in Tasker (2010), IECV2 results were tested in Allegra (2017), IECV3 results were tested in Friis Petersen (2019), IECV4 results were tested in Jayaprakasan (2010) , IECV5 results were tested in Klinkert (2005), IECV6 results were tested in Lan (2013), IECV7 results were tested in Lefebvre (2015), IECV8 results were tested in Magnusson (2017), IECV9 results were tested in Olivennes (2015), IECV10 results were tested in Oudshoorn (2017), IECV11 results were tested in Popovic-Todorovic (2003), IECV12 results were tested in Van Tilborg (2017), IECV13 results were tested in Bastu (2016), IECV14 results were tested in Klinkert (unpublished).


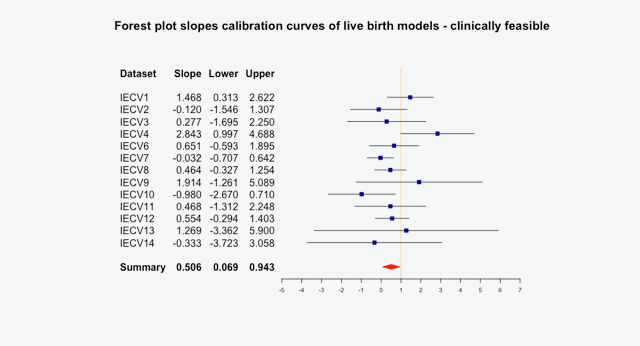


Forest plot of slopes of the clinically feasible live birth models including age, starting dose and anti-Müllerian hormone. IECV = internal external cross-validation set, Lower = lower 95% of confidence interval, Upper = upper 95% of confidence interval. Summary statistic results in an overall slope of 0.506 (0.069-0.943). IECV1 results were tested in Tasker (2010), IECV2 results were tested in Allegra (2017), IECV3 results were tested in Friis Petersen (2019), IECV4 results were tested in Jayaprakasan (2010) , IECV5 results were tested in Klinkert (2005), IECV6 results were tested in Lan (2013), IECV7 results were tested in Lefebvre (2015), IECV8 results were tested in Magnusson (2017), IECV9 results were tested in Olivennes (2015), IECV10 results were tested in Oudshoorn (2017), IECV11 results were tested in Popovic-Todorovic (2003), IECV12 results were tested in Van Tilborg (2017), IECV13 results were tested in Bastu (2016), IECV14 results were tested in Klinkert (unpublished).


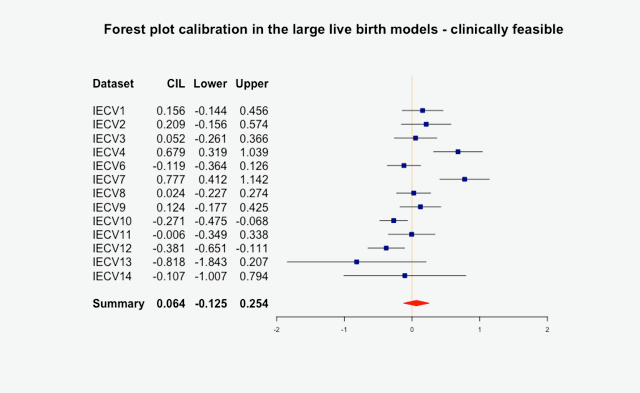


Forest plot of calibration in the large of the live birth models including age, starting dose and anti-Müllerian hormone. IECV = internal external cross-validation set, Lower = lower 95% of confidence interval, CIL = calibration in the large Upper = upper 95% of confidence interval. Summary statistic results in an overall calibration in the large of 0.064 (-0.125 – 0.254). IECV1 results were tested in Tasker (2010), IECV2 results were tested in Allegra (2017), IECV3 results were tested in Friis Petersen (2019), IECV4 results were tested in Jayaprakasan (2010) , IECV5 results were tested in Klinkert (2005), IECV6 results were tested in Lan (2013), IECV7 results were tested in Lefebvre (2015), IECV8 results were tested in Magnusson (2017), IECV9 results were tested in Olivennes (2015), IECV10 results were tested in Oudshoorn (2017), IECV11 results were tested in Popovic-Todorovic (2003), IECV12 results were tested in Van Tilborg (2017), IECV13 results were tested in Bastu (2016), IECV14 results were tested in Klinkert (unpublished).

Calibration in the large & slope of calibration curves Safety (optimal)


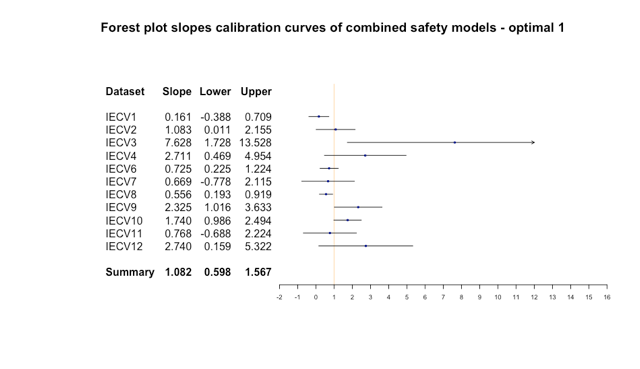


Forest plot of slopes of the optimal safety model 1 including age, starting dose, anti-Müllerian hormone and basal follicle stimulating hormone. IECV = internal external cross-validation set, Lower = lower 95% of confidence interval, Upper = upper 95% of confidence interval. Summary statistic results in an overall slope of 1.082 (0.598-1.567). IECV1 results were tested in Tasker (2010), IECV2 results were tested in Allegra (2017), IECV3 results were tested in Friis Petersen (2019), IECV4 results were tested in Jayaprakasan (2010) , IECV5 results were tested in Klinkert (2005), IECV6 results were tested in Lan (2013), IECV7 results were tested in Lefebvre (2015), IECV8 results were tested in Magnusson (2017), IECV9 results were tested in Olivennes (2015), IECV10 results were tested in Oudshoorn (2017), IECV11 results were tested in Popovic-Todorovic (2003), IECV12 results were tested in Van Tilborg (2017), IECV13 results were tested in Bastu (2016), IECV14 results were tested in Klinkert (unpublished).


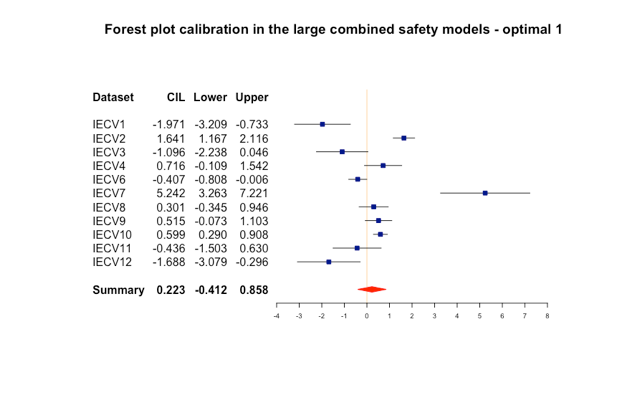


Forest plot of calibration in the large of the optimal safety model 1 including age, starting dose, anti-Müllerian hormone and basal follicle stimulating hormone. IECV = internal external cross-validation set, Lower = lower 95% of confidence interval, CIL = calibration in the large, Upper = upper 95% of confidence interval. Summary statistic results in an overall calibration in the large of 0.223 (-0.412 – 0.858). IECV1 results were tested in Tasker (2010), IECV2 results were tested in Allegra (2017), IECV3 results were tested in Friis Petersen (2019), IECV4 results were tested in Jayaprakasan (2010) , IECV5 results were tested in Klinkert (2005), IECV6 results were tested in Lan (2013), IECV7 results were tested in Lefebvre (2015), IECV8 results were tested in Magnusson (2017), IECV9 results were tested in Olivennes (2015), IECV10 results were tested in Oudshoorn (2017), IECV11 results were tested in Popovic-Todorovic (2003), IECV12 results were tested in Van Tilborg (2017), IECV13 results were tested in Bastu (2016), IECV14 results were tested in Klinkert (unpublished).


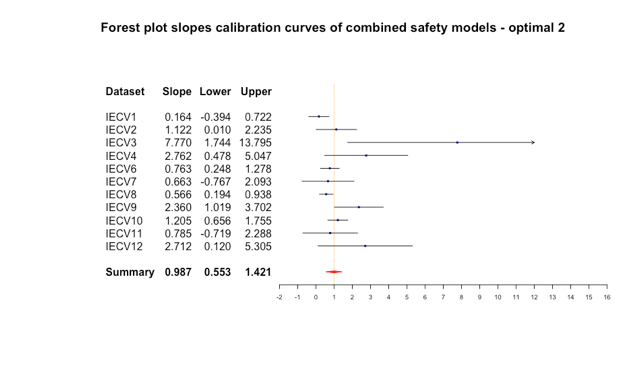


Forest plot of slopes of the optimal safety model 2 including age, starting dose, anti-Müllerian hormone, basal follicle stimulating hormone and GnRH-cotreatment. IECV = internal external cross-validation set, Lower = lower 95% of confidence interval, Upper = upper 95% of confidence interval. Summary statistic results in an overall slope of 0.987 (0.553-1.421). IECV1 results were tested in Tasker (2010), IECV2 results were tested in Allegra (2017), IECV3 results were tested in Friis Petersen (2019), IECV4 results were tested in Jayaprakasan (2010) , IECV5 results were tested in Klinkert (2005), IECV6 results were tested in Lan (2013), IECV7 results were tested in Lefebvre (2015), IECV8 results were tested in Magnusson (2017), IECV9 results were tested in Olivennes (2015), IECV10 results were tested in Oudshoorn (2017), IECV11 results were tested in Popovic-Todorovic (2003), IECV12 results were tested in Van Tilborg (2017), IECV13 results were tested in Bastu (2016), IECV14 results were tested in Klinkert (unpublished).


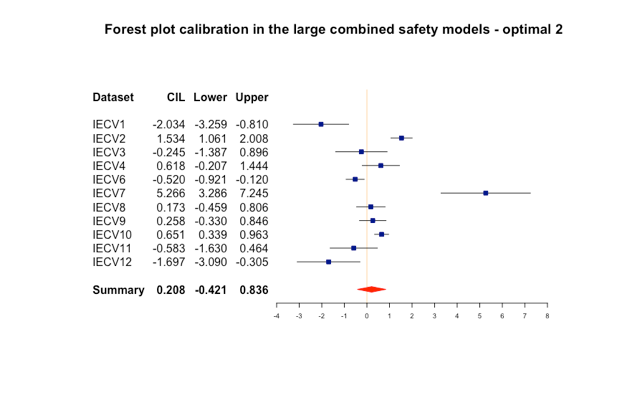


Forest plot of calibration in the large of the optimal safety model 2 including age, starting dose, anti-Müllerian hormone, basal follicle stimulating hormone and GnRH-cotreatment. IECV = internal external cross-validation set, Lower = lower 95% of confidence interval, Upper = upper 95% of confidence interval. Summary statistic results in an overall AUC of 0.208 (-0.421-0.836). IECV1 results were tested in Tasker (2010), IECV2 results were tested in Allegra (2017), IECV3 results were tested in Friis Petersen (2019), IECV4 results were tested in Jayaprakasan (2010) , IECV5 results were tested in Klinkert (2005), IECV6 results were tested in Lan (2013), IECV7 results were tested in Lefebvre (2015), IECV8 results were tested in Magnusson (2017), IECV9 results were tested in Olivennes (2015), IECV10 results were tested in Oudshoorn (2017), IECV11 results were tested in Popovic-Todorovic (2003), IECV12 results were tested in Van Tilborg (2017), IECV13 results were tested in Bastu (2016), IECV14 results were tested in Klinkert (unpublished).

Calibration in the large & slope of calibration curves - Safety (clinically feasible)


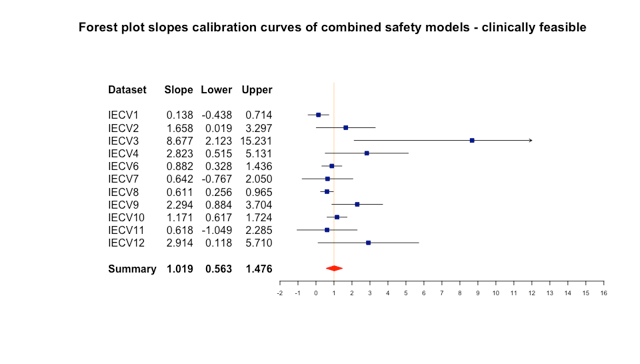


Forest plot of slopes of the clinically feasible safety model including age, starting dose, anti-Müllerian hormone and GnRH-cotreatment. IECV = internal external cross-validation set, Lower = lower 95% of confidence interval, Upper = upper 95% of confidence interval. Summary statistic results in an overall slope of 1.019 (0.563-1.476). IECV1 results were tested in Tasker (2010), IECV2 results were tested in Allegra (2017), IECV3 results were tested in Friis Petersen (2019), IECV4 results were tested in Jayaprakasan (2010) , IECV5 results were tested in Klinkert (2005), IECV6 results were tested in Lan (2013), IECV7 results were tested in Lefebvre (2015), IECV8 results were tested in Magnusson (2017), IECV9 results were tested in Olivennes (2015), IECV10 results were tested in Oudshoorn (2017), IECV11 results were tested in Popovic-Todorovic (2003), IECV12 results were tested in Van Tilborg (2017), IECV13 results were tested in Bastu (2016), IECV14 results were tested in Klinkert (unpublished).


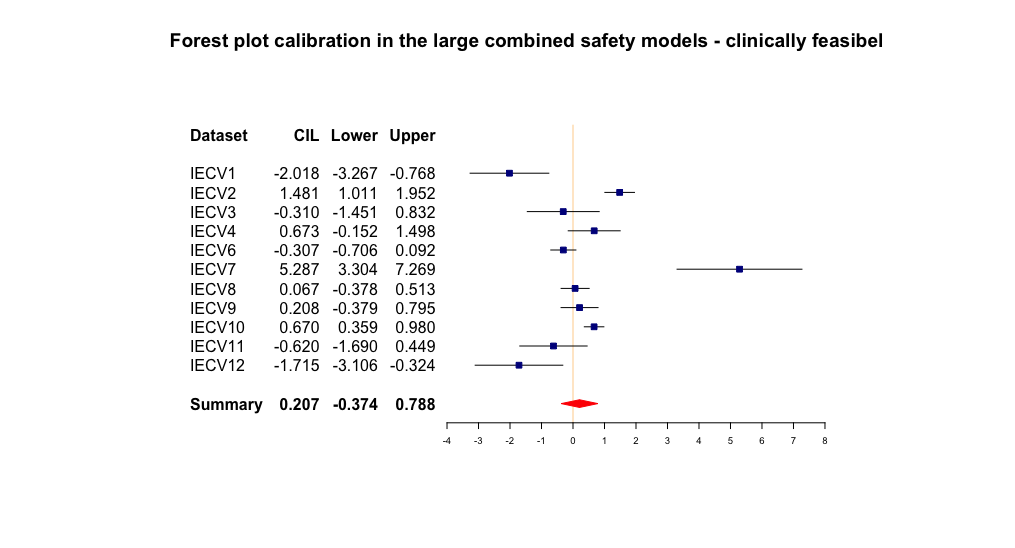


Forest plot of calibration in the large of the clinically feasible safety model including age, starting dose, anti-Müllerian hormone and GnRH-cotreatment. IECV = internal external cross-validation set, Lower = lower 95% of confidence interval, CIL = calibration in the large, Upper = upper 95% of confidence interval. Summary statistic results in an overall calibration in the large of 0.207 (-0.374 – 0.788). IECV1 results were tested in Tasker (2010), IECV2 results were tested in Allegra (2017), IECV3 results were tested in Friis Petersen (2019), IECV4 results were tested in Jayaprakasan (2010) , IECV5 results were tested in Klinkert (2005), IECV6 results were tested in Lan (2013), IECV7 results were tested in Lefebvre (2015), IECV8 results were tested in Magnusson (2017), IECV9 results were tested in Olivennes (2015), IECV10 results were tested in Oudshoorn (2017), IECV11 results were tested in Popovic-Todorovic (2003), IECV12 results were tested in Van Tilborg (2017), IECV13 results were tested in Bastu (2016), IECV14 results were tested in Klinkert (unpublished).

Calibration plots of internal validation prediction models


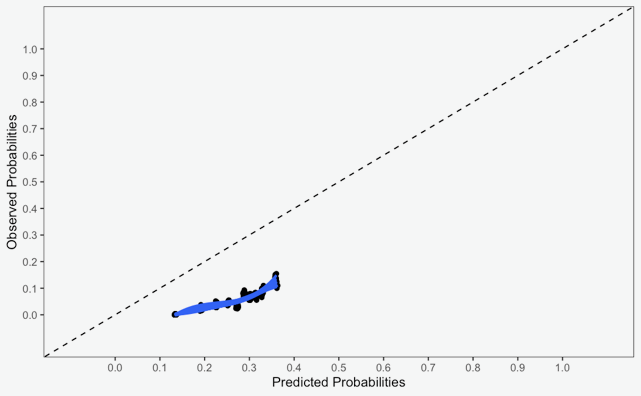


Calibration plot of final optimal live birth model. The slope of this calibration curve is 2.463 with a calibration in the large of -1.838. A perfect slope would be 1 and a perfect calibration in the large 0. On the x-axis are the predicted probabilities and on the y-axis are the observed probabilities.


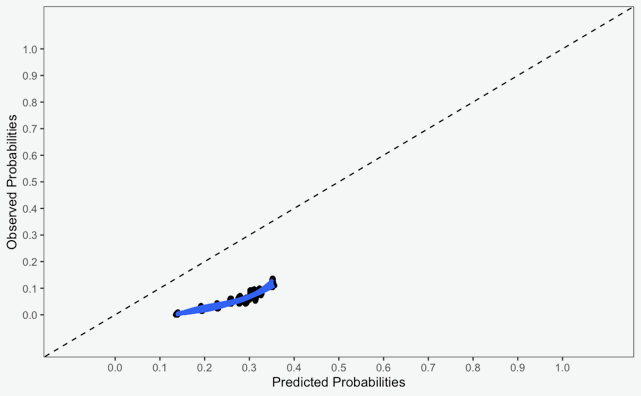


Calibration plot of final clinically feasible live birth model. The slope of this calibration curve is 2. 475 with a calibration in the large of -1. 840. A perfect slope would be 1 and a perfect calibration in the large 0. On the x-axis are the predicted probabilities and on the y-axis are the observed probabilities.


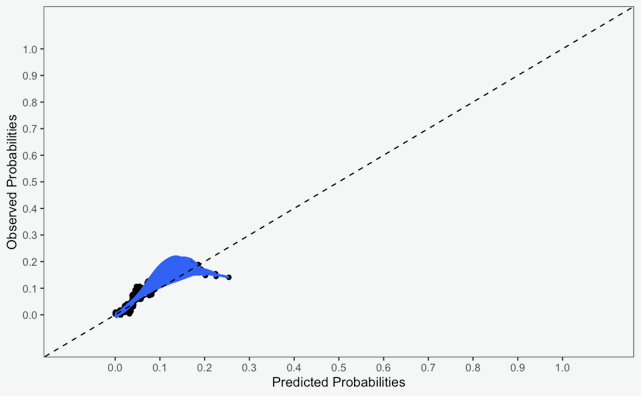


Calibration plot of final optimal combined treatment risk model 1. The slope of this calibration curve is 0.848 with a calibration in the large of -0.158. A perfect slope would be 1 and a perfect calibration in the large 0. On the x-axis are the predicted probabilities and on the y-axis are the observed probabilities.


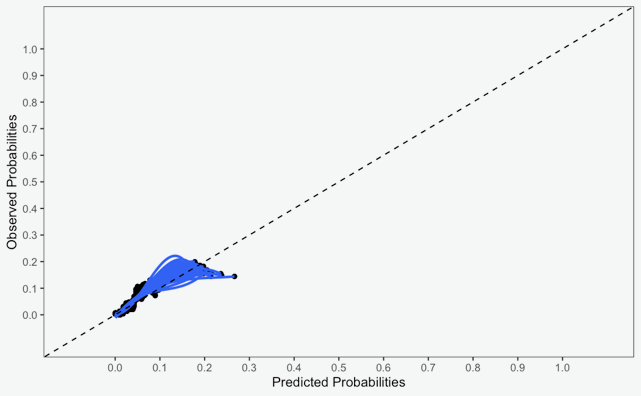


Calibration plot of final optimal combined treatment risk model 2. The slope of this calibration curve is 0.842 with a calibration in the large of -0.119. A perfect slope would be 1 and a perfect calibration in the large 0. On the x-axis are the predicted probabilities and on the y-axis are the observed probabilities.


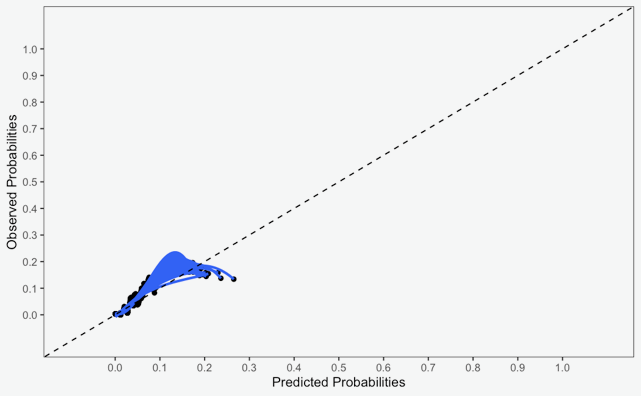


Calibration plot of final clinically feasible combined treatment risk model. The slope of this calibration curve is 0.856 with a calibration in the large of -0.131. A perfect slope would be 1 and a perfect calibration in the large 0. On the x-axis are the predicted probabilities and on the y-axis are the observed probabilities.

Starting dose and age effect of prediction models


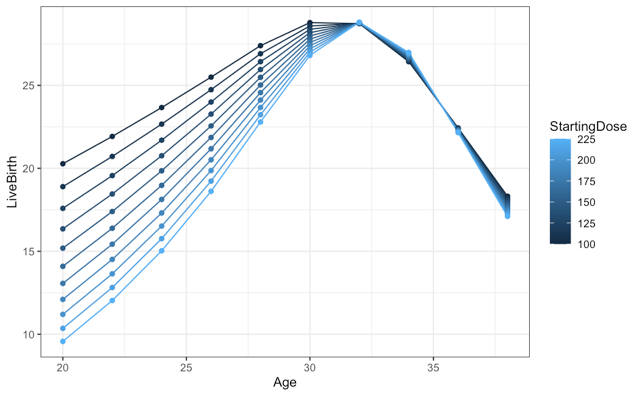


Plot visualizing the relationship between age, starting dose and live birth for the optimal live birth prediction model. On the y-axis the chance of live birth (in percentage) is shown, on the x-axis age in years. The different plotted lines indicate starting dose in IU. This is plotted for a patient with an AMH of 3.2 ng/mL, AFC of 15 follicles 2-10 mm, bFSH of 7.3 IU/L, centerdose 0 and centerage 0, and GnRH agonist. These specific values were chosen as they are the means of the dataset. For younger women, adjusting the starting dose has an effect on their live birth chance. Starting from 30 years, chances of live birth decline with an increasing age. However, starting dose does not influence live birth chances in women older than 30 years.


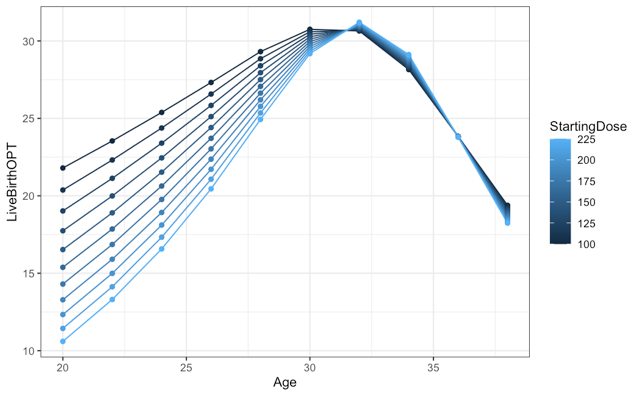


Plot visualizing the relationship between age, starting dose and live birth for the clinically feasible live birth prediction model. On the y-axis the chance of live birth (in percentage) is shown, on the x-axis age in years. The different plotted lines indicate starting dose in IU. This is plotted for a patient with an AMH of 3.2 ng/mL, AFC of 15 follicles 2-10 mm, bFSH of 7.3 IU/L, centerdose 0 and centerage 0, and GnRH agonist. These specific values were chosen as they are the means of the dataset. For younger women, adjusting the starting dose has an effect on their live birth chance. Starting from 30 years, chances of live birth decline with an increasing age. However, starting dose does not influence live birth chances in women older than 30 years.


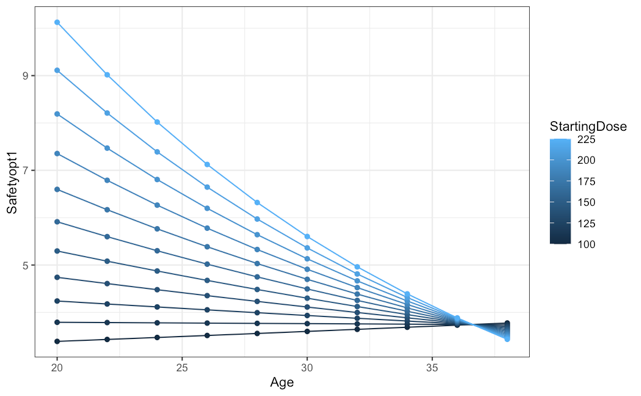


Plot visualizing the relationship between age, starting dose and combined treatment risk the optimal prediction model 1. On the y-axis the treatment risk (in percentage) is shown, on the x-axis age in years. The different plotted lines indicate starting dose in IU. This is plotted for a patient with an AMH of 3.2 ng/mL, AFC of 15 follicles 2-10 mm, bFSH of 7.3 IU/L, centerdose 0 and centerage 0, and GnRH agonist. These specific values were chosen as they are the means of the dataset. For younger women, adjusting the starting dose has the most effect on their treatment risk. A lower starting dose results in a lower treatment risk. With increasing age, this effect declines, as does the risk of having a safety event.


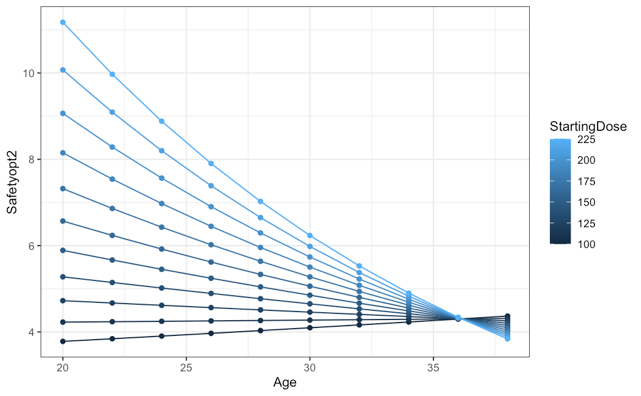


Plot visualizing the relationship between age, starting dose and combined treatment risk the optimal prediction model 2. On the y-axis the treatment risk (in percentage) is shown, on the x-axis age in years. The different plotted lines indicate starting dose in IU. This is plotted for a patient with an AMH of 3.2 ng/mL, AFC of 15 follicles 2-10 mm, bFSH of 7.3 IU/L, centerdose 0 and centerage 0, and GnRH agonist. These specific values were chosen as they are the means of the dataset. For younger women, adjusting the starting dose has the most effect on their treatment risk. A lower starting dose results in a lower treatment risk. With increasing age, this effect declines, as does the risk of having a safety event.


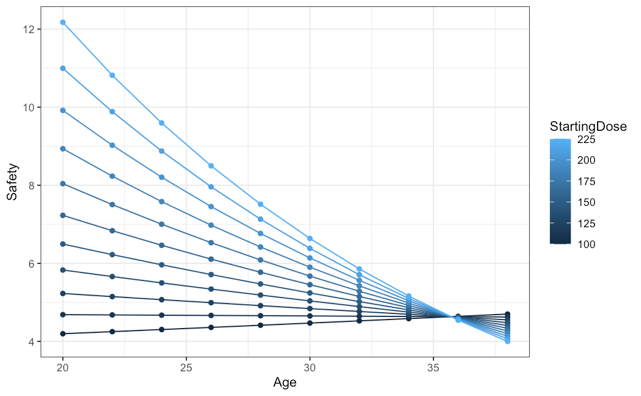


Plot visualizing the relationship between age, starting dose and combined treatment risk the clinically feasible prediction model. On the y-axis the treatment risk (in percentage) is shown, on the x-axis age in years. The different plotted lines indicate starting dose in IU. This is plotted for a patient with an AMH of 3.2 ng/mL, AFC of 15 follicles 2-10 mm, bFSH of 7.3 IU/L, centerdose 0 and centerage 0, and GnRH agonist. These specific values were chosen as they are the means of the dataset. For younger women, adjusting the starting dose has the most effect on their treatment risk. A lower starting dose results in a lower treatment risk. With increasing age, this effect declines, as does the risk of having a safety event.
